# Effects of habitats in typical karst areas of Guizhou on ultrastructural morphology of Typhlocybinae

**DOI:** 10.1002/ece3.10680

**Published:** 2023-11-05

**Authors:** Jiajia Chen, Jia Jiang, Ni Zhang, Yuehua Song

**Affiliations:** ^1^ School of Karst Science Guizhou Normal University Guiyang China; ^2^ Engineering Technology Institute for Karst Desertification Control Guiyang China

**Keywords:** karst, sensilla, Typhlocybinae, ultrastructure

## Abstract

Biology and the environment are inextricably linked. Insects are intricately linked to their habitats as part of the ecosystem. In this study, scanning electron microscopy (SEM) observations showed two sensilla chaetica, several sensilla trichodea, and at least one sensilla basiconicum on the antennae of typhlocybine insects. There were no sensilla on the labrum; however, the surface morphology was different. There were more sensilla trichodea on the surface, mostly symmetrically distributed along the labial groove, and there was little difference in the structure of the stylet fascicle. The correlation between the sensilla number on the body surface of typhlocybine insects and environmental factors in the 3 study areas was as follows: Huajiang > Bijie > Shibing, which is consistent with the classification of rocky desertification grade; that is, the higher the rocky desertification grade, the greater the sensilla number on leafhoppers affected by the environment. The correlation between the number of leafhoppers and environmental factors in the 3 months was as follows: end of September > end of May > end of July, which was consistent with the changing temperature trends. The results of this study enrich our knowledge of the morphological characteristics of leafhoppers and explore the potential value of insect surface ultra‐morphology for use by humans.

## Graphical abstract

The exploration of the morphological characteristics of leafhoppers is essential. Most studies focus have rarely been on the interrelationships between leafhoppers and the environment. The ultrastructure of the leafhopper body surface and scientific issues are summarized.

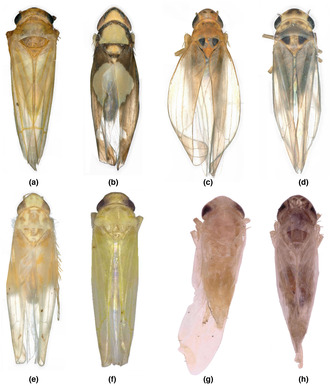

## 1 INTRODUCTION

Typhlocybine insects belong to Cicadellidae, Auchenorrhyncha, and Hemiptera and are the second largest group of Cicadellidae after Deltocephalinae. Generally, Typhlocybinae is divided into six groups according to the venation differences between fore and hind wings: Alebrini, Dikraneurini, Empoascini, Erythroneurini, Typhlocybini, and Zyginellini; many scholars have conducted relevant classification research (Dworakowska, 1977, 1979; Mahmood, 1967; Matsumura, 1931; Metcalf, 1968). According to the World Auchenorrhyncha Database (https://proceps.github.io/auchenorrhyncha/#/), there are approximately 5071 valid species in 522 genera distributed across six zoogeographic regions worldwide (Dmitrieva et al., n.d.). The insects in this group are small (2–5 mm) and numerous. They are minimally migratory and spread phytoplasma‐associated diseases in a continuous reproductive manner (Hirao & Inoue, 1979; Renaudin et al., 2015). They use their mouthparts to pierce host tissues and extract the juice of host plants, which can cause white spots on leaves, and in severe cases, leaf wilting and shedding (Backus & McLean, 1982; Leopold et al., 2003).

Ultrastructure is determined by scanning a sample using scanning electron and transmission electron microscopes to emit extremely fine high‐energy electron beams and perform secondary electron imaging of the ultra‐micromorphological structure of insects using the point‐by‐point imaging method. The antenna is the sensory organ of the insect head and plays an extremely important role in insect communication, host location, feeding, mate seeking, reproduction, habitat, defense, and migration (Liu, Xiao, & Chen, 2020; Na et al., 2008a). Schneider (1964) was the first to describe in detail insect antennae and the sensilla on the surface of the antennae and pointed out that many types of sensilla are attached to the antennae. Mouthparts, as the main feeding organs of insects, have differentiated between a variety of feeding methods and various types of mouthparts over long‐term evolution (Smith & Capinera, 2005; Snodgrass, 1935). Insect mouthparts have a high differentiation rate. In paleontology studies, researchers have subdivided insect mouthparts into 34 living types and two fossil types (Labandeira, 1997; Liu et al., 2005). Leafhoppers have pierce‐sucking mouthparts composed of the labrum, labium, and stylet fascicle (Jin, 2012; Leopold et al., 2003; Pan, 2013; Zhao et al., 2010). Pollard (1972) conducted a detailed study on the structure of the mandibular and maxillary stylets of *Eupteryx melissae* (Curtis, 1837), which was the first detailed report on the stylet of a leafhopper (Pollard, 1972). Subsequently, some researchers studied the mouthpart morphology and feeding patterns of leafhoppers (Backus & McLean, 1982; Leopold et al., 2003; Tavella & Arzone, 1993). Available data suggest that the details of insect antennae and mouthpart morphology, including shape, segmentation, and fine structure, differ across taxa, and these differences can be used to differentiate taxa (Brożek, 2006; Brożek & Bourgoin, 2013a, 2013b; Liu, Xiao, & Chen, 2020).

Over the long history of evolution, organisms have been closely related to the environment and have formed an inseparable whole. The environment affects organisms through matter and energy, and organisms are constantly affecting and adapting to the environment. Insects can sense changes in the external environment through their body surface sensilla and find suitable behavioral sites by identifying volatile compounds released by plants (Bruce & Pickett, 2011). For instance, as phytophagous insects, leafhoppers feed and jump on plant leaves and sense the external environment through mechanical and chemical sensilla on their body's surface (Altner & Loftus, 1985). Considering that sensilla trichodea (ST), multiporous peg‐like sensilla (PGSM), dome‐shaped sensilla (DSSM), and other sensilla can sense external temperature (Brożek & Bourgoin, 2013), it is speculated that the number of sensilla on the body surface of typhlocybine insects are more during the high‐temperature season to better perceive the external temperature and to prevent evaporation of water in the insect itself and sunburn on the body surface.

Thus far, research on insect body surface ultrastructure and the environment has mostly focused on the relationship between volatile components and insect body surface ultrastructure. There have been few studies on other environmental factors and insect body surface ultrastructure. Therefore, in this study, after investigating the biodiversity of Typhlocybinae in different karst habitats, scanning electron microscopy was used to observe the mouthparts and antennae of Typhlocybinae. Combined with its habitat characteristics, the similarities and differences in the ultrastructure of the same species of Typhlocybinae under different karst habitat conditions and the similarities and differences in the ultrastructure of different typhlocybine species under the same environmental conditions were analyzed, and their correlation with the environment was explored.

## 2 MATERIALS AND METHODS

### 2.1 Study areas

**FIGURE 1:**
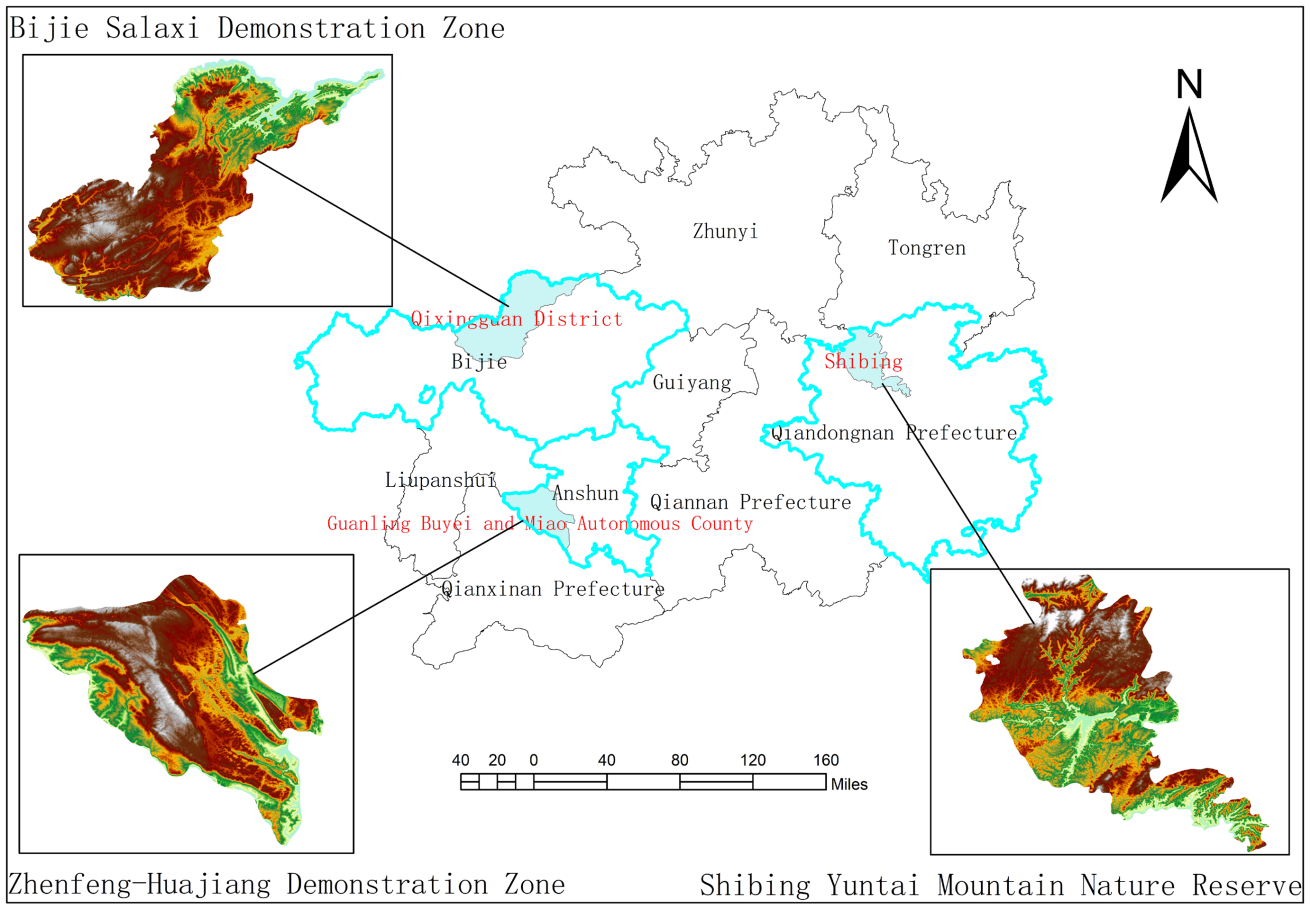
Distribution of three research areas in Guizhou.

Guizhou Province is located in the eastern part of the Yunnan–Guizhou Plateau in southwestern China. It is located at (103°36′–109°35′ E, 24°37′–29°13′ N), with an altitude range of (147.8–2900.6 m; Figure 1). It has a humid subtropical monsoon climate with abundant annual rainfall. The average annual precipitation is 1100–1300 mm (Chen et al., 2017). The fragile ecological environment in this region is widely distributed, and soil erosion is a severe problem (Zhao et al., 2020). After an in‐situ investigation, three typical karst demonstration areas with apparent differences in geographical environments were selected as study areas: the Zhenfeng‐Huajiang Demonstration Zone (Huajiang), Bijie Salaxi Demonstration Zone (Bijie), and Shibing Yuntai Mountain Nature Reserve (Shibing). The three study areas represent different habitats and are located at a certain geographical distance, all of which avoid areas with intense human disturbance.

According to the classification standard of the rocky desertification intensity grade in karst areas, Xiong et al. (2002) divided the rocky desertification grade into six grades (Table 1). The three study areas were classified as follows: Huajiang (moderate to severe rocky desertification), Bijie (light to moderate rocky desertification), and Shibing (without potential rocky desertification) (Xiong et al., 2002).

**TABLE 1:** Grading standard of rocky desertification intensity in the Karst area.

Degree of rocky desertification	Percentage of exposed rock/%	Soil coverage/%	Slope/(°)	Coverage of vegetation and soil/%	Average of soil thickness/cm
No obvious rocky desertification (ND)	<20	>60	<15	>80	>20
Potential rocky desertification (PD)	20–30	<60	>15	70–80	<20
Light rocky desertification (LD)	31–50	<30	>18	50–69	<15
Moderate rocky desertification (MD)	51–70	<20	>22	30–49	<10
Strong rocky desertification (SD)	71–90	<10	>25	10–29	<5
Extremely strong rocky desertification (ESD)	>90	<5	>30	<10	<3

**FIGURE 2:**
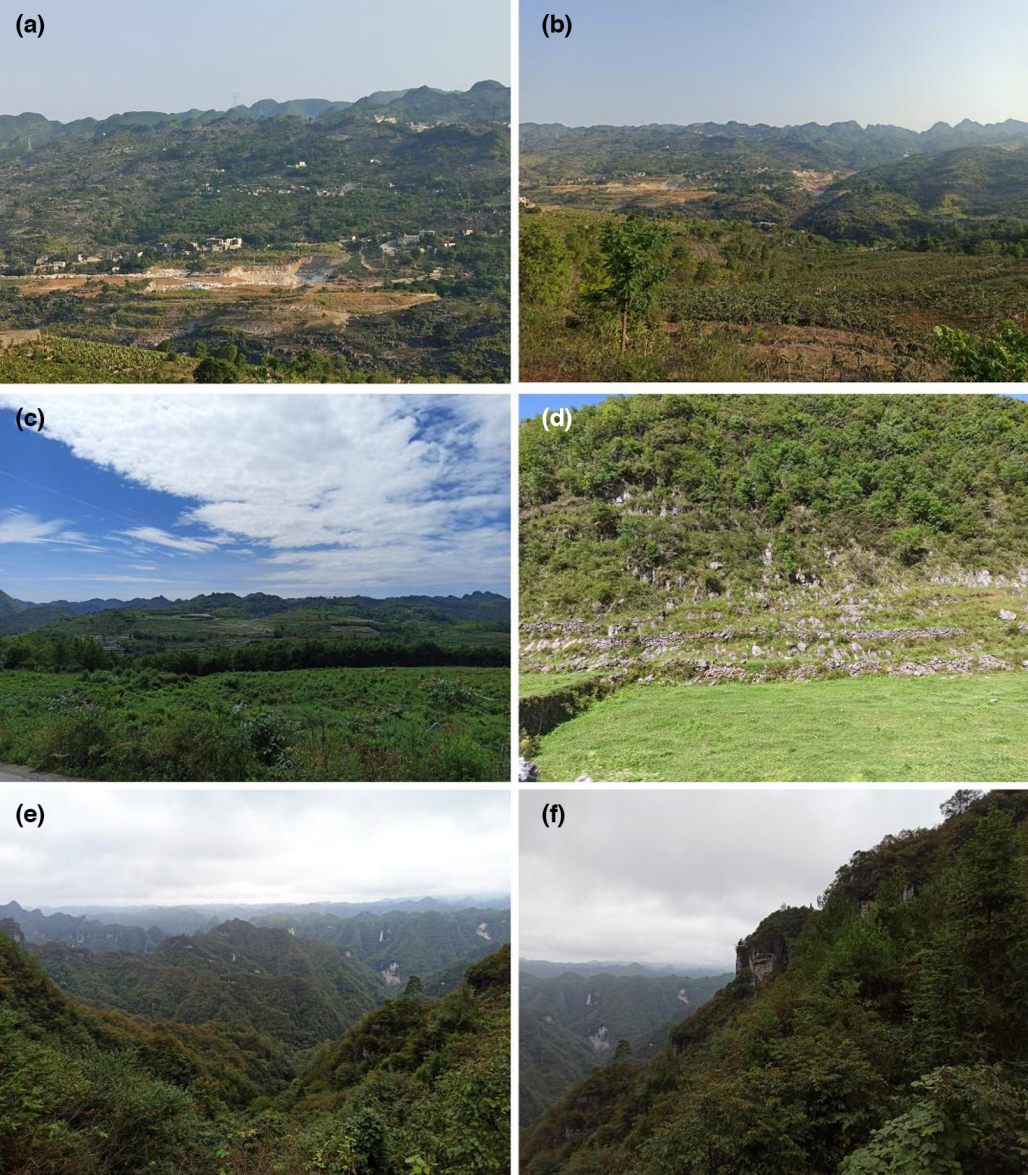
Habitat characteristics in three study areas. (a, b) Huajiang. (c, d) Bijie. (e, f) Shibing.

The Zhenfeng‐Huajiang Demonstration Zone (105°34′59″–105°43″06″ E, 25°37′18″–25°42′37″ N) is located along the Beipan River in southwestern Guizhou Province. The altitude is 400–1400 m. It has a tropical subtropical dry‐hot valley climate, with high temperatures and low humidity. In this area, the soil layer is shallow, the vegetation coverage is low, and large areas of carbonate rock are exposed, which further leads to soil erosion and vegetation degradation. Low vegetation coverage and biomass were observed in the study area (Figure 2a,b).

The Bijie Salaxi Demonstration Zone (105°01′11″–105°08′38″ E, 27°11′09″–27°17′28″ N) is located in the Liuchong River Basin, Qixingguan District, northwestern Guizhou Province. The altitude is approximately 1500–2200 m. It has a northern subtropical monsoon humid climate with low temperatures, severe weather, such as heavy snow and hail, high altitude, weak evaporation, and little difference in precipitation from the Huajiang study area. The terrain in the area fluctuates greatly, and after the vegetation has been destroyed, the soil is lost and accumulates in depressions. Because of the lower air temperature and weaker transpiration, the leaf water content is slightly higher than that in the other two study areas (Figure 2c,d).

The Shibing Yuntai Mountain Nature Reserve (108°01′36″–108°10′52″ E, 27°13′56″–27°04′51″ N) is located in the upper middle part of the Wuyang River Basin in Shibing County, central‐eastern Guizhou Province. The altitude is approximately 600–1250 m. It has a subtropical humid monsoon climate with high temperatures, large seasonal changes, high humidity, more precipitation days, and the largest amount of precipitation of the three areas. As a World Natural Heritage Site, the Yuntai Mountains in Shibing have been well protected. This area has deep soil, lush vegetation, high vegetation coverage, and tall trees (Figure 2e,f).

### 2.2 Collection of specimens

Based on the vegetation types in the three study areas, five host plants were selected: *Juglans regia* (Linnaeus, 1753), *Rhus chinensis* (Miller, 1768), *Prunus persica* (Linnaeus, 1753), *Prunus salicina* (Lindley, 1830), and *Debregeasia orientalis* (Chen, 1991). The focal leafhopper species of five host plants in different study sites were collected (Table S1). One sample plot was selected for each host plant from each study area (Table 2). Samples were collected from the study areas at the end of September 2020, May 2021, and July 2021. Based on the phototaxis of the leafhoppers, samples were collected using the sweeping net method when the weather was fine. A collecting net with a diameter of 30 cm was swept back and forth on the host plant. Specimens were collected once every 60 sweeps. Each study plot was swept three times for a total of 180 times, and the collected samples were stored in 2.5% glutaraldehyde. Six to fifteen healthy and undamaged leaves growing on the sunny side of the plant (the number of leaves sampled depended on their size) were selected as plant specimens and stored in a sealed bag. Samples were placed in an ice box below 5°C, taken back to the laboratory, and immediately placed in a refrigerator at 4°C for later use. After each sample collection, a label was assigned to record the geographic coordinates, temperature, humidity, altitude, and vegetation type of the sample plot.

**TABLE 2:** Overview of the sample plots.

Study areas	Host plant	Altitude (m)	Latitude	Longitude
Huajiang	*Juglans regia*	1200	25°42′29″ N	105°36′50″ E
	*Rhus chinensis*	1285	25°41′36″ N	105°37′46″ E
	*Prunus persica*	1193	25°40′43″ N	105°38′57″ E
	*Prunus salicina*	1190	25°37′43″ N	105°41′8″ E
	*Debregeasia orientalis*	1240	25°39′56″ N	105°38′1″ E
Bijie	*Juglans regia*	1960	27°14′5″ N	105°5′51″ E
	*Rhus chinensis*	1760	27°14′43″ N	105°6′16″ E
	*Prunus persica*	1959	27°15′49″ N	105°5′13″ E
	*Prunus salicina*	1766	27°14′51″ N	105°5′52″ E
	*Debregeasia orientalis*	1700	27°13′25″ N	105°5′47″ E
Shibing	*Juglans regia*	950	27°2′39″ N	108°7′23″ E
	*Rhus chinensis*	952	27°2′53″ N	108°7′25″ E
	*Prunus persica*	1000	27°12′48″ N	108°11′5″ E
	*Prunus salicina*	1140	27°13′35″ N	108°1′54″ E
	*Debregeasia orientalis*	912	27°7′51″ N	108°2′27″ E

### 2.3 Climate data collection

The temperature data were obtained from field measurements. A thermometer was used to measure the highest and lowest daily air temperature. The average air temperature was calculated as follows: (highest air temperature + lowest air temperature)/2. The amount of precipitation was obtained from meteorological stations in the region, and the number of precipitation days was obtained from field inspection records.

### 2.4 Blade measurement data

Six leaves of similar size were collected between 12:00 and 14:00 when the plant photosynthesis was the strongest. Leaf length, maximum leaf width, and leaf thickness were measured using Vernier calipers, and the fresh weight (FW) of the leaves was measured using an electronic balance. The methodological steps for dry weight (DW) were as follows: place the leaves in an oven at 105°C for 30 min, dry them at 70°C to a constant weight, remove them, and weigh the DW. The formula for calculating leaf water content (LWC) is (Luo et al., 2012):

$$\mathrm{LWC}\;\left(\%\right)=\frac{\mathrm{FW}-\mathrm{DW}}{\mathrm{FW}}\times 100\%$$

### 2.5 Specimen classification and identification

**FIGURE 3:**
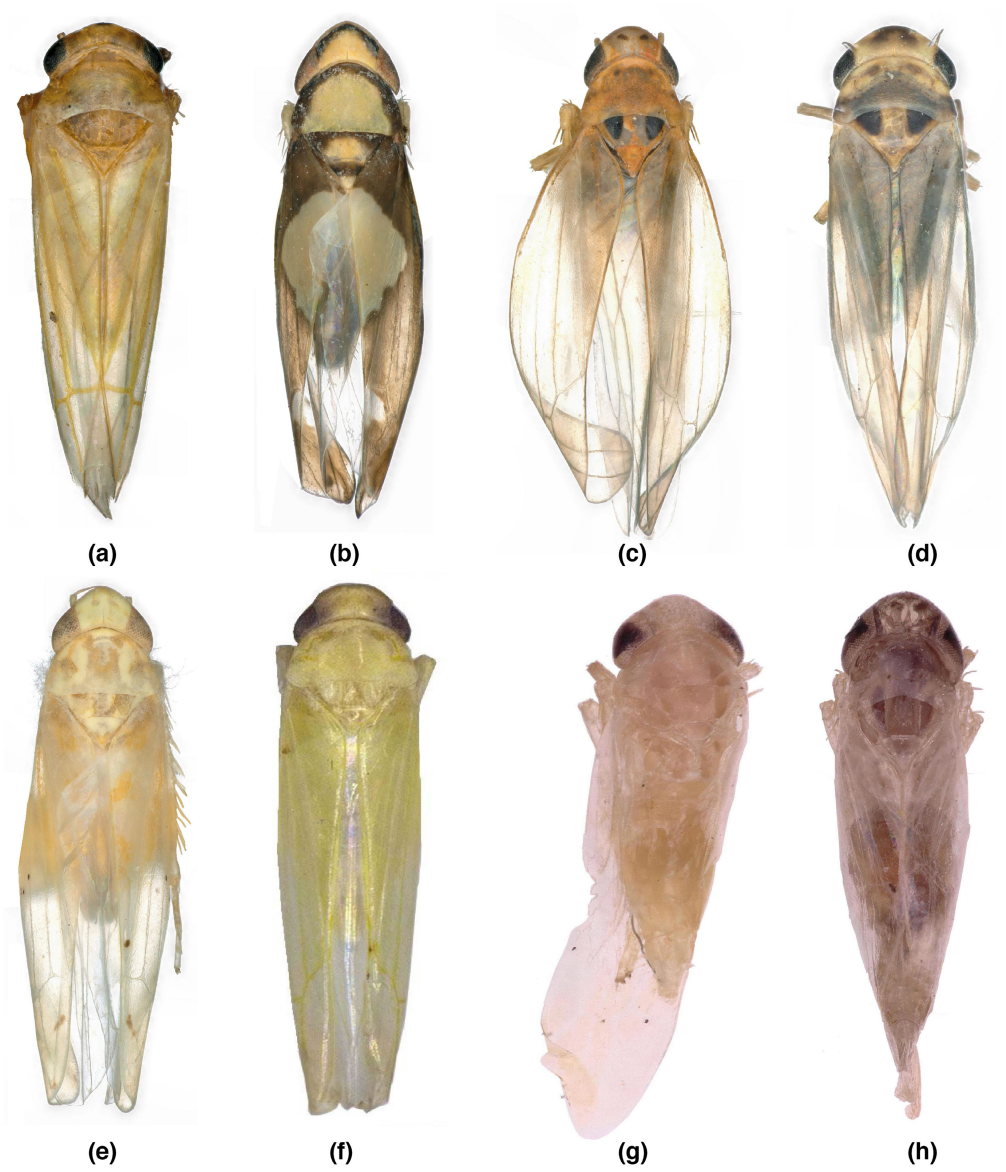
Habitus of eight leafhopper species in dorsal view (a: *Singapora shinshana*, b: *Zyginella minuta*, c: *Arboridia remmi*, d: *Empoascanara sipra*, e: *Limassolla lingchuanensis*, f: *Alnetoidia dujuanensis*, g: *Asymmetrasca rybiogon*, h: *Empoasca* sp. ‐9).

Leafhoppers were removed from the refrigerator and roughly classified according to venation type and external characteristics. Then, the male specimens were removed, and the abdomen was soaked in a 5%–10% NaOH solution and heated in a water bath for 8–15 min. After cooling, the samples were washed with water and dissected under an Olympus stereoscope using a dissecting needle. After dissection, the genitals were stored in glycerol. According to the characteristics of male genitalia, species identification is based on 3I (3I Interactive Keys and Taxonomic Databases) (http://dmitriev.speciesfile.org/) and the following references: “Erythroneurini and Zgyinellini in China (Homoptera: Cicadellidae: Typhlocybinae)”, “Pictorial of Insect Type Specimens Deposited in Guizhou University”, “Taxonomic Study on Chinese Empoascini (Homoptera: Cicadellidae)”, “Systematic Study of Typhlocybini from China (Homoptera: Cicadellidae: Typhlocybinae)”, and “Systematics study on Tribes Alebrini and Dikraneurini from China” (Dmitry, n.d.; Huang, 2003; Jiao, 2017; Li et al., 2014; Qin, 2003; Song & Li, 2014). Professor Song Yuehua, a leafhopper taxonomist, helped with identification. The leafhopper samples were identified as containing the following eight species (Figure 3): *Singapora shinshana* (Matsumura, 1932), *Zyginella minuta* (Yang, 1965), *Arboridia remmi* (Vilbaste, 1968), *Empoascanara sipra* (Dworakowska, 1980), *Limassolla lingchuanensis* (Chou & Zhang, 1985), *Alnetoidia dujuanensis* (Song & Li, 2010), *Asymmetrasca rybiogon* (Dworakowska, 1971), and *Empoasca* sp. ‐9. All specimens were deposited in the School of Karst Science, Guizhou Normal University (GZNU), China.

### 2.6 Scanning electron microscopy (SEM) samples

The widespread and dominant species in the study areas were selected, and three well‐preserved male specimens were selected and dissected under an Olympus SZX16 stereo microscope. The head and wings of the adult were removed, and the surface was dried with filter paper and then washed with phosphate buffer saline (PBS, 0.1 M, pH 7.2) five times, 5 min each time. The samples were shaken in an ultrasonic cleaner for 30 s (except for the wings) and then dehydrated in 30%, 50%, 70%, 90%, 95%, and 100% acetonitrile solutions for 20 min. The samples were then attached to aluminum stubs using double‐sided tape. The samples were sputtered with gold/palladium using a GVC‐2000 ion sputtering instrument and then observed and photographed using a QUANTA FEG 250 SEM at 20 kV.

### 2.7 Temperature and humidity

**FIGURE 4:**
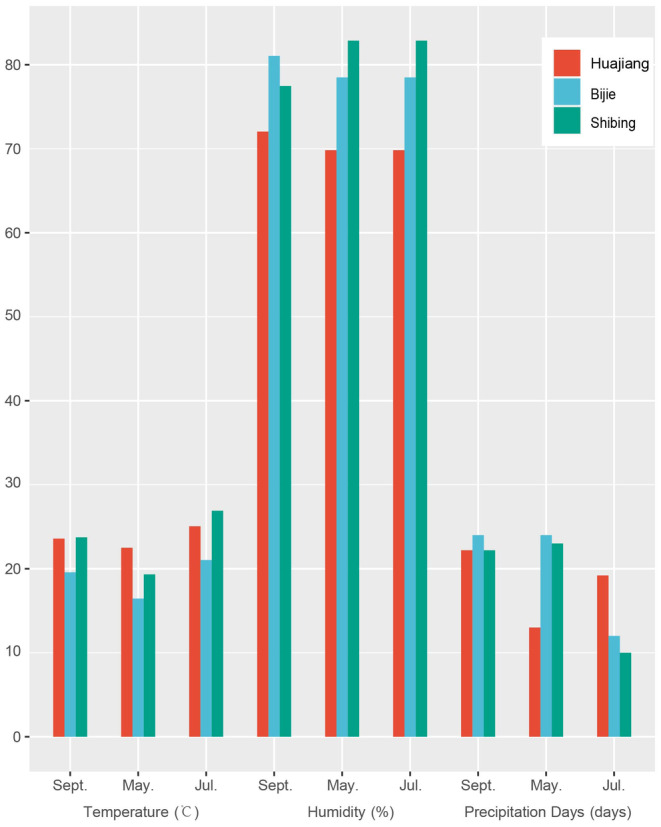
Climate characteristics in three study areas.

Based on the climate data for September 2020, and May and July 2021 for the three study areas (Table S2), the climatic characteristics of these 3 months were obtained by averaging (Figure 4). The monthly average temperature changes in the three study areas were as follows: July > September > May. Temperature changes in the Huajiang study area were smaller during the different months, and the temperature changes in Bijie and Shibing were relatively consistent. The temperature in the Bijie study area was the lowest, and the average annual temperatures of the other two areas were relatively similar. The humidity in the three study areas was notably different, and the changes in different months were also different. There is lush vegetation and strong plant transpiration, which makes the change in atmospheric humidity small in the Shibing study area, while the Huajiang study area has sparse vegetation and shallow soil layers. The surface runoff was rapid, the soil retained less water, the temperature was high, and the water evaporated rapidly, resulting in a large change in atmospheric humidity. The rainy days in Bijie and Shibing were relatively similar in terms of numerical values and trends. There were more rainy days in September and May; rainfall in September was relatively large, and the number of precipitation days in July was low. There were notable differences in the Huajiang study area; there were fewer precipitation days in May, and more rainy days in July and September.

### 2.8 Host plants

**FIGURE 5:**
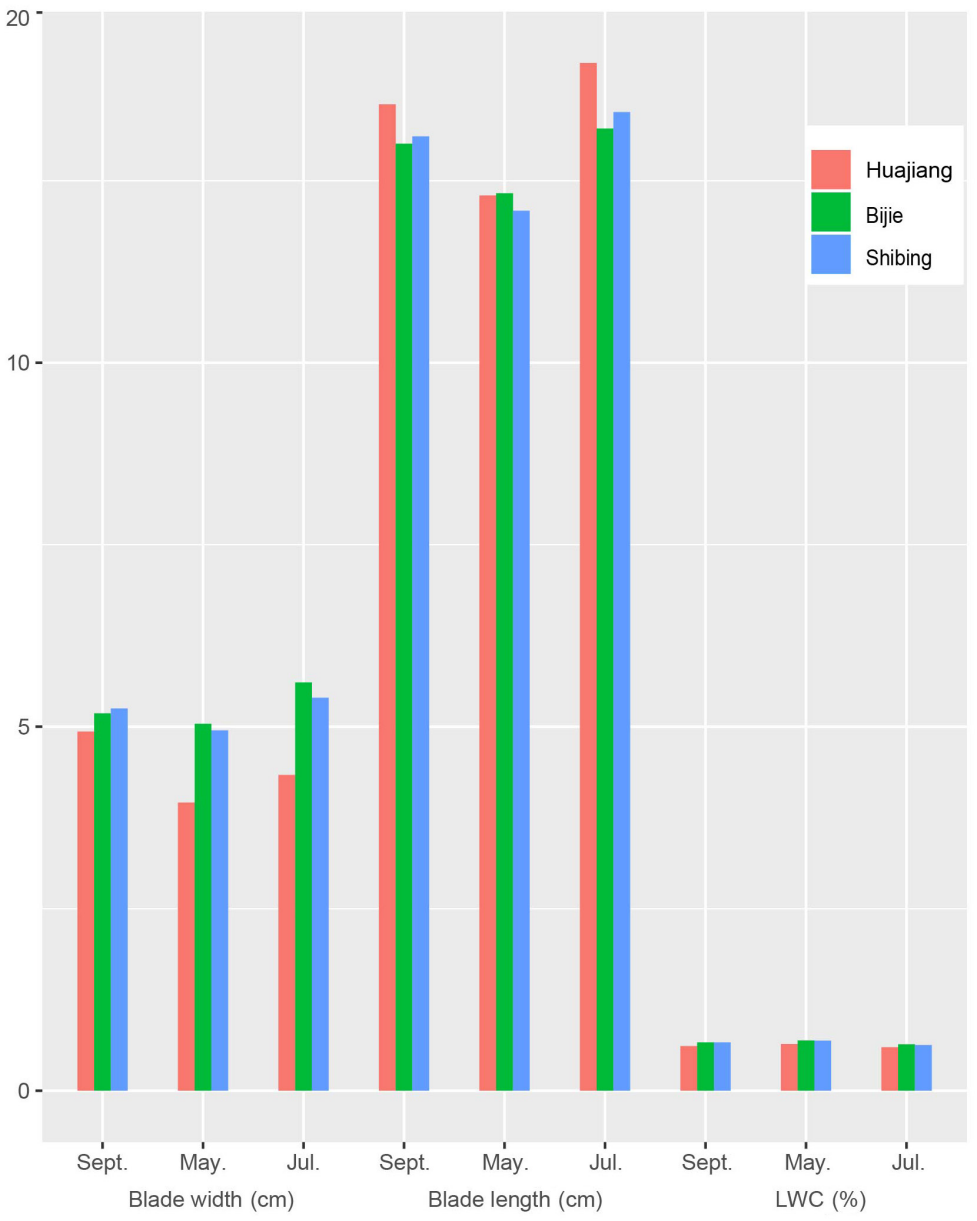
Leaf characteristics of host plants in three study areas (LWC, Leaf water content).

According to the host plant leaf data for each month in the study areas (Table S3), the average value of each plot was taken as the host plant leaf characteristics for these 3 months (Figure 5). The variation trends of plant leaf length in the three study areas were consistent as follows: July > September > May, and the leaf length of plants in May was significantly shorter. The host plants in the Huajiang study area had longer leaf lengths and smaller leaf widths, whereas the leaf widths and leaf lengths of the host plants in the other two study areas were relatively similar, and the leaf surface area was the largest in July and the smallest in May. There was little difference in the water content of the host plants in the three study areas, and this followed the order May > September > July, which was negatively correlated with temperature changes in the study areas.

### 2.9 Data processing

The best‐preserved leafhopper individuals in the samples were selected to count the number of sensilla, and a redundancy analysis (RDA) was performed in combination with environmental factors. The sensilla chaetica on the scape (Sc‐SC), sensilla trichodea on the pedicel (Pe‐ST), sensilla basiconica on the fiagellum (Fl‐SB), sensilla trichodea on the first section of the labium (Lb1‐ST), sensilla trichodea on the second section of the labium (Lb2‐ST), sensilla trichodea on the third section of the labium (Lb3‐ST), sensilla basiconica on the third section of the labium (Lb3‐SB), and the cone‐like sensilla on the third section of the labium (Lb3‐SB), as well as the habitat characteristics (temperature (T), humidity (H), days of precipitation (PD), host plant blade length (BL), blade width (BW), and leaf water content (LWC)) in the study areas were used to establish the main factors influencing the number of surface sensilla of typhlocybine leafhoppers. Data analysis was performed and visualized using Microsoft Excel 2016 and Canoco 5 (Cronk, 2016; Mahajan & Gokhale, 2019; Šmilauer & Lepš, 2014), Figures 4 and 5 were conducted by R (v4.3.1) with the package “ggplot2”.

## 3 RESULTS

### 3.1 Habitat and body surface ultrastructure

A total of 4078 Typhlocybine specimens, belonging to 121 species in 41 genera and six tribes, were collected (Table S4). At the study area level, the number of individuals was as follows: Shibing > Bijie > Huajiang, and the number of different taxon levels was Bijie (6 tribes, 32 genera, 68 species) > Huajiang (6 tribes, 22 genera, 58 species) > Shibing (5 tribes, 21 genera, 48 species). The distribution by time was as follows: July > May > September, and the number of different levels of taxa was May (6 tribes, 29 genera, 61 species) > September (6 tribes, 21 genera, 64 species) > July (4 tribes, 21 genera, 64 species).

**FIGURE 6:**
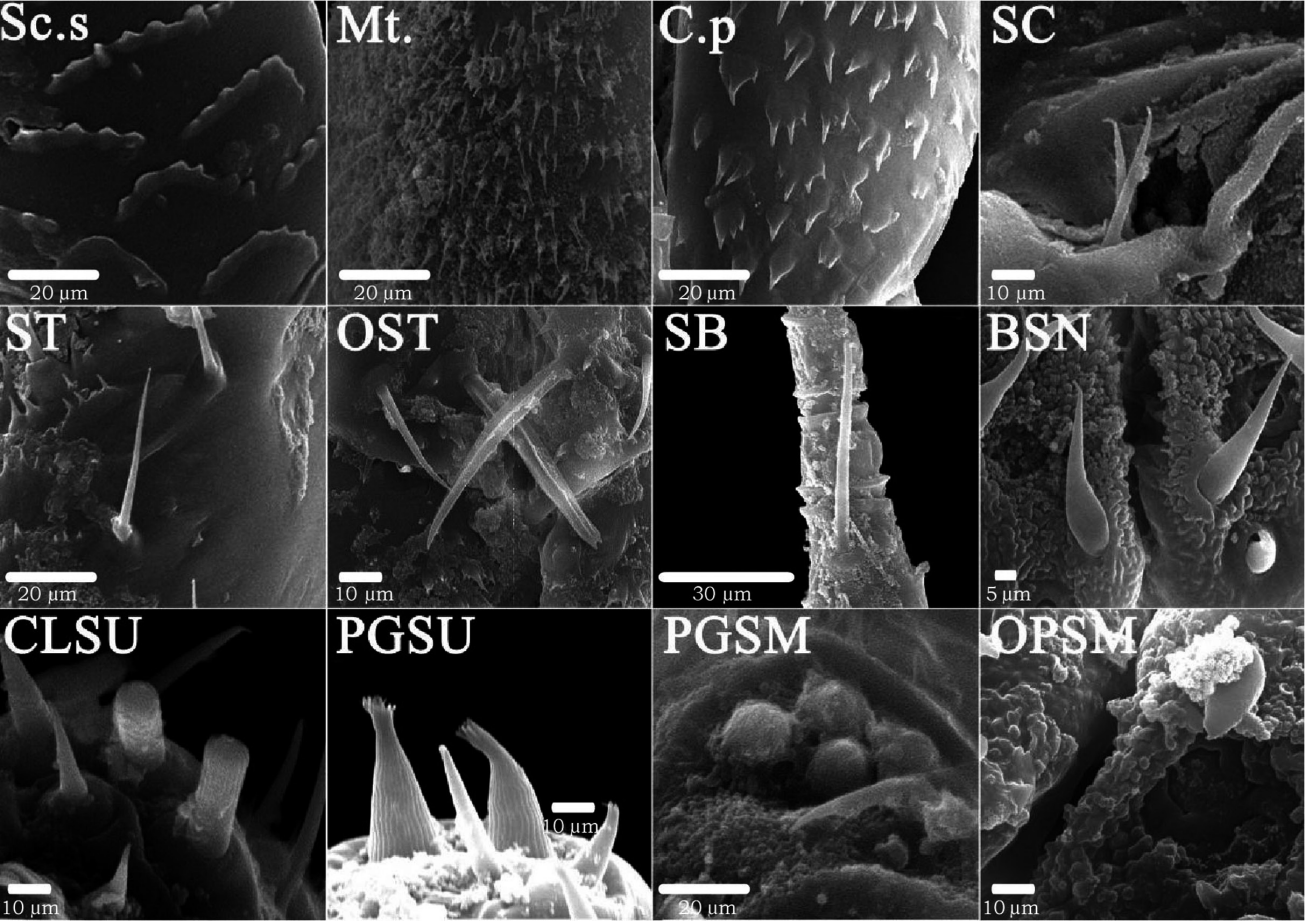
Types of sensilla and epidermal protrusions.

According to the samples collected in the field, from the two dimensions of time and space, male typhlocybine specimens with wide distribution, sufficient quantity, and well‐preserved morphological structure were selected (Table S5). After processing, the male leafhopper specimens were photographed using a scanning electron microscope, the average length data of the samples were selected (Table S6), and the number of sensilla from the most well‐preserved individuals in the samples was counted (Table S7). Three types of epidermal protrusions and nine types of sensilla were observed on all sample surfaces (Figure 6). The three epidermal protrusions included scaly structures (Sc.s): fish‐scale protrusions; microtrichia (Mt): fine villous, and cuticular processes (C.p): triangular. The nine different types of sensilla included sensilla chaetica (SC): smooth surface, extremely pointed end; sensilla trichodea (ST): hairy, curved, or slightly curved; oversized sensilla trichodea (OST): large, hairy, curved, or slightly curved; sensilla basiconica (SB): conical, shorter, and thicker; cone‐like sensilla (BSN): the front end is thicker and the end is sharper; clavate‐like sensilla (CLSU): rod‐like, shorter, and thicker; uniporous peg‐like sensilla (PGSU): nail‐shaped; PGSM: eggshell‐like; oval plate sensilla (OPSM): oval‐shaped (Sensilla nomenclature based on: (Aljunid & Anderson, 1983; Brożek & Bourgoin, 2013a; Hao et al., 2016; Stacconi & Romani, 2012; Zhang et al., 2020)).

#### 3.1.1 Antenna ultrastructure

The antennae of typhlocybine insects are located on the face. They are setaceous antennae consisting of three parts: scape, pedicel, and flagellum. The overall length of antennae in this sample ranged from 650 to 1150 μm, with certain interspecific differences. The length relationships of the three parts were as follows: flagellum > pedicel > scape (Table S6). The ultrastructure of the antennal epidermal protrusions of different species had certain interspecific differences but tended to be consistent within species. The types of sensilla on the antennae of all species were the same; only the number differed. However, the pattern of change was not obvious.

**FIGURE 7:**
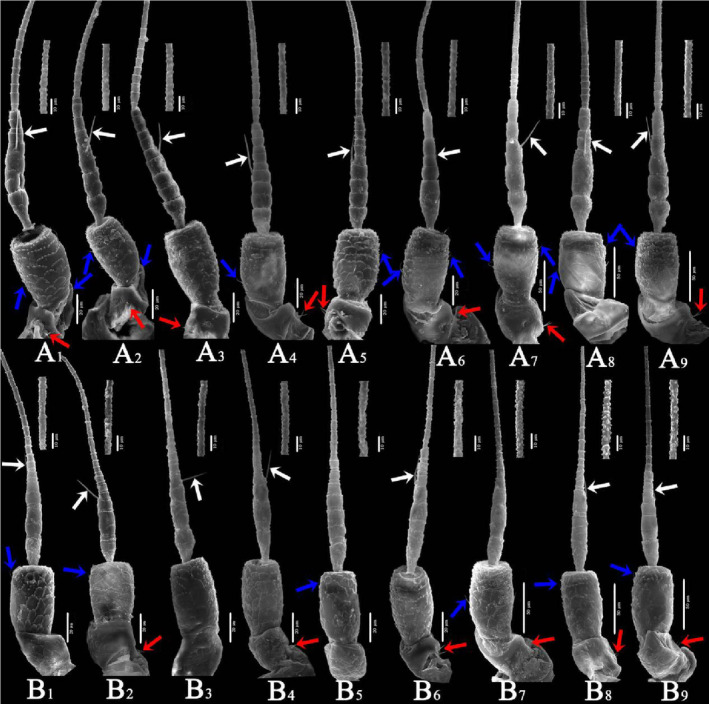
SEM observation of the antennae of Typhlocybinae species (White arrow: SB; Blue arrow: ST; Red arrow: SC) (a: *Singapora shinshana*; b: *Zyginella minuta*; 1, 2, 3….9: Different samples of the same species).

**FIGURE 8:**
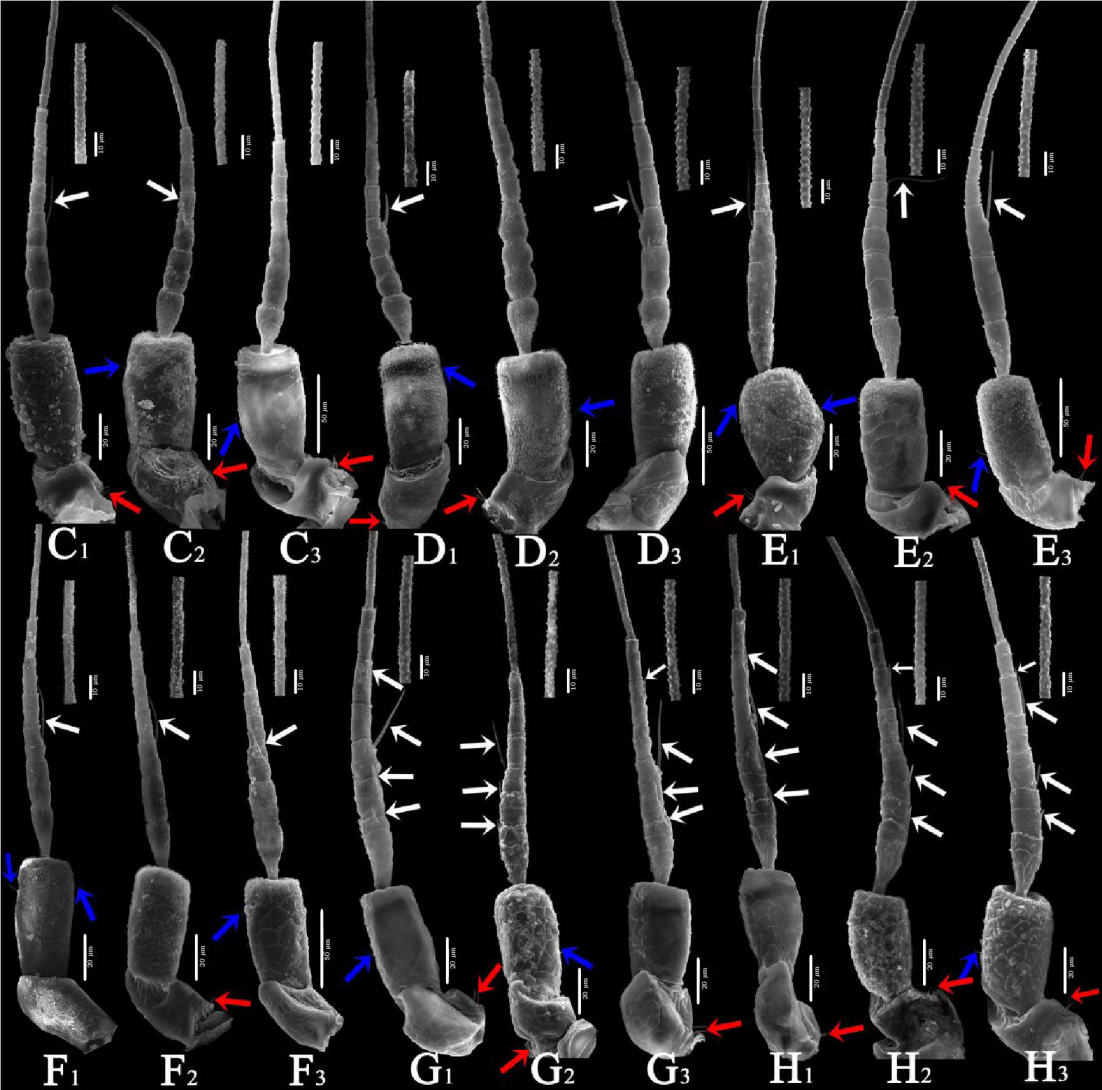
SEM observation of the antennae of Typhlocybinae species (Continual figure of Figure 7) (White arrow: SB; Blue arrow: ST; Red arrow: SC) (c: *Arboridia remmi*; d: *Empoascanara sipra*; e: *Limassolla lingchuanensis*; f: *Alnetoidia dujuanensis*; g: *Asymmetrasca rybiogon*; h: *Empoasca* sp. ‐9; 1, 2, 3: Different samples of the same species).

In general (Figures 7 and 8), the ultrastructure of the antennal integumental protrusions of different species showed certain interspecific differences but tended to be consistent within the species (Table 3). From the overall morphology of the antennae, the morphological characteristics of the antennae of two species of the tribe Zyginellini (*Z. minuta* and *L. lingchuanensis*) and two species of the tribe Empoascini (*A. rybiogon* and *E*. sp. ‐9) were very similar, whereas those of the other four erythroneurine leafhoppers were quite different in morphology.

**TABLE 3:** Comparison of the ultrastructure of antennae of 8 leafhopper species.

Species	Morphology						
	Scape		Pedicel		Flagellum		Antenna total length (μm)
	Length (μm)	Sensilla type (number)	Length (μm)	Sensilla type (number)	Length (μm)	Sensilla type (number)	
*S. shinshana*	49.47	SC (2)	81.94	Sc.s/ST (3–5)	591.53	Mt./SB (1)/HP	650–800
*Z. minuta*	42.56	Sc.s/SC (2)	72.91	Sc.s/ST (2–3)	584.47	Mt./Sc.s/SB (1)/HP	650–750
*A. remmi*	56.21	SC (2)	89.30	Sc.s/ST (2–4)	767.29	Mt. /SB (1)/SP	800–1050
*E. sipra*	45.86	Mt./Sc.s/SC (2)	77.00	Mt./Sc.s/ST (3–5)	622.47	Mt./SB (2)/HP	700–800
*L. lingchuanensis*	37.02	SC (2)	81.00	Sc.s/ST (3–4)	635.04	Mt./Sc.s/SB (1)/HP	700–800
*A. dujuanensis*	68.18	Mt./Sc.s/SC (2)	96.07	Mt./Sc.s/ST (3–4)	862.83	Mt./SB (1)	1000–1050
*A. rybiogon*	51.89	Mt./Sc.s/SC (2)	83.99	Sc.s/ST (3–4)	936.54	SB (4)/SP	1000–1050
*E*. sp. ‐9	46.80	Sc.s/SC (2)	80.19	Sc.s/ST (3–4)	917.98	Sc.s/SB (4)/HP	1000–1100

Abbreviations: HP, hemispheric protrusion; SP, spherical protrusion.

In *L. lingchuanensis* and *A. rybiogon*, which also feed on *Rhus chinensis*, although their pedicle lengths and sensilla types were very similar, the lengths, sensilla types, and numbers varied between the scape and flagellum, likely because *A. rybiogon* sample collection was broad and included more complex habitat conditions. *Z. minuta* and *A. dujuanensis* also feed on *Juglans regia*, although *A. dujuanensis* has a greater advantage in terms of antennal length and number of sensilla. However, the types of sensilla of *Z. minuta* were more abundant, possibly because the scanning samples of *Z.minuta* came from different regions and months; therefore, it has a wealth of sensilla to help adapt to a variety of complex environments. Although *S. shinshana*, *A. remmi*, and *E. sipra* originate from different host plants, the overall morphology of their antennae is relatively similar, probably because of the similarity in their biological characteristics.

All species had both sensilla trichodea and sensilla basiconica on their antennae but no other types of sensilla. These sensilla differ not only in number but also in type. The morphology of the antennae is mainly affected by kinship and less by the environment. In the present study, all typhlocybine leafhoppers had setae‐like antennae with elongated ends. Unlike Pentatomidae insects, there are obvious differences in the morphology of antennae terminals, some of which are spherical, some flat, and some cone‐shaped (Rani & Madhavendra, 2005).

#### 3.1.2 Ultrastructure of mouthparts

**FIGURE 9:**
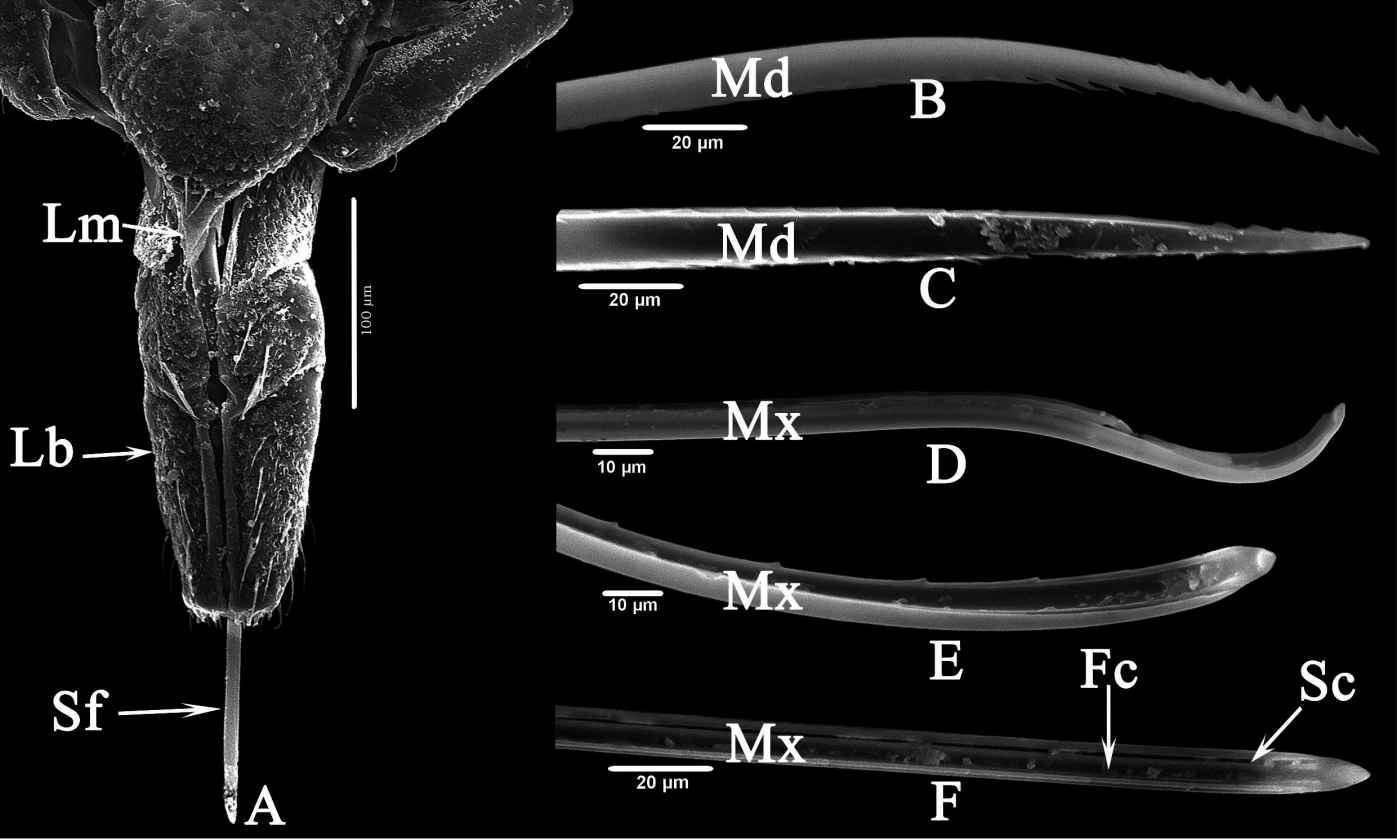
SEM observation of the mouthparts of Typhlocybine species. (a) Overall view of Stylet fascicle. (b) Dorsal view of Mandibular stylet. (c) Ventral view of Mandibular stylet. (d) Dorsal view of Maxillary stylet. (e) Ventral view of Maxillary stylet. (f) Ventral view of Maxillary stylet, showing Fc and Sc.

The mouthparts of typhlocybine insects are typical piercing‐sucking mouthparts that penetrate the host plant to suck the sap. The mouthparts are composed of the labrum (Lm), labium (Lb), and stylet fascicle (Sf). The stylet fascicle is composed of mandibular stylets (Md) and maxillary stylets (Mx). The labrum is triangular and covers the cylindrical labium. The labium is divided into three sections, which are divided into left and right parts by the labial groove at the center. The stylet fascicle is enclosed in the labial groove, is elongated and comprises a pair of discrete mandibular and chimeric maxillary stylets. The base is connected to the labium and the two maxillary stylets form two hollow thin tubes through interlocking and tight fitting. The thick food canal (Fc) is located in the middle, and a thin salivary canal (Sc) is adjacent to the food canal (Figure 9).

**FIGURE 10:**
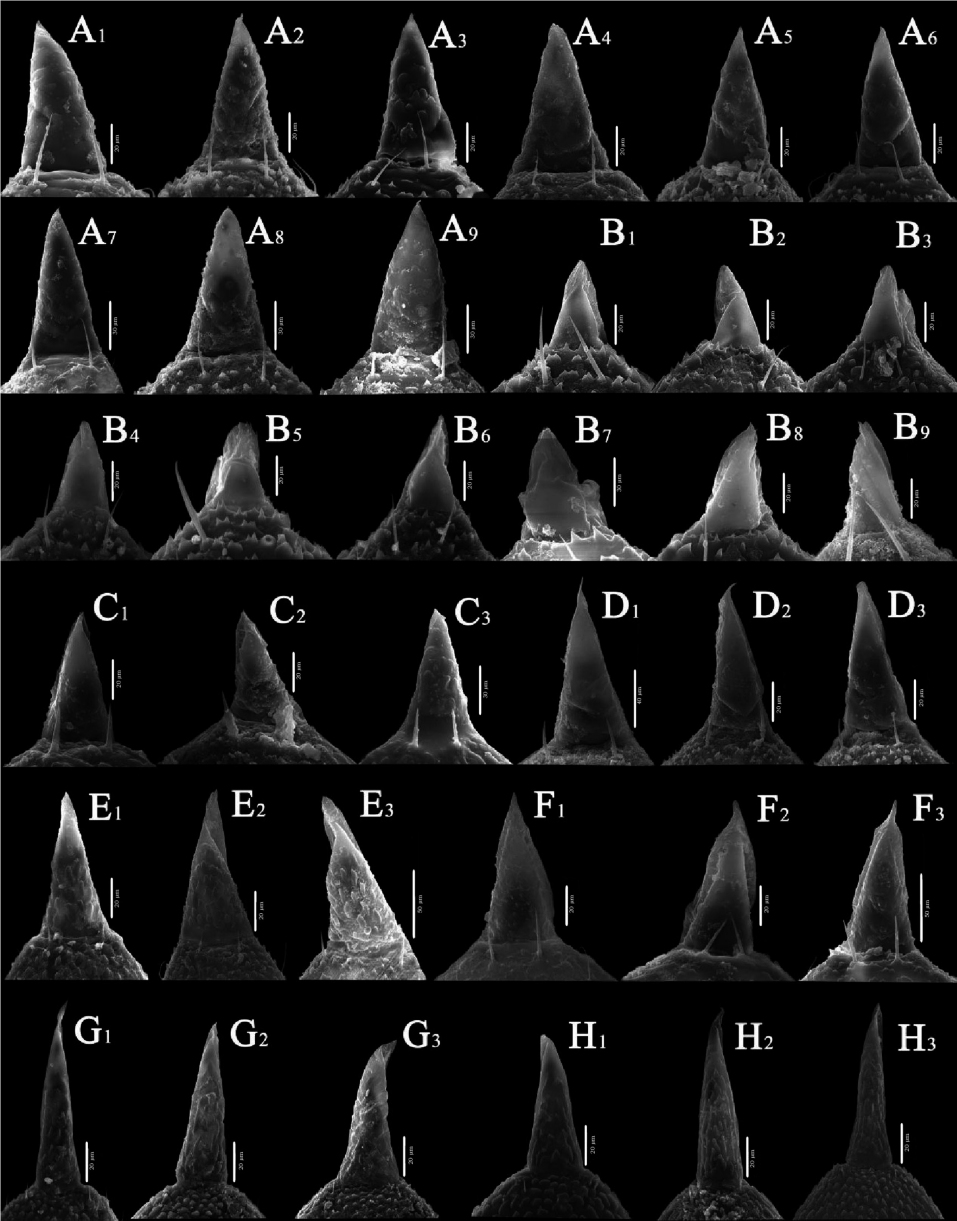
SEM observation of the labrum (Lm) of Typhlocybine species (a: *Singapora shinshana*; b: *Zyginella minuta*; c: *Arboridia remmi*; d: *Empoascanara sipra*; e: *Limassolla lingchuanensis*; f: *Alnetoidia dujuanensis*; g: *Asymmetrasca rybiogon*; h: *Empoasca* sp. ‐9; 1, 2, 3….9: Different samples of the same species).

**FIGURE 11:**
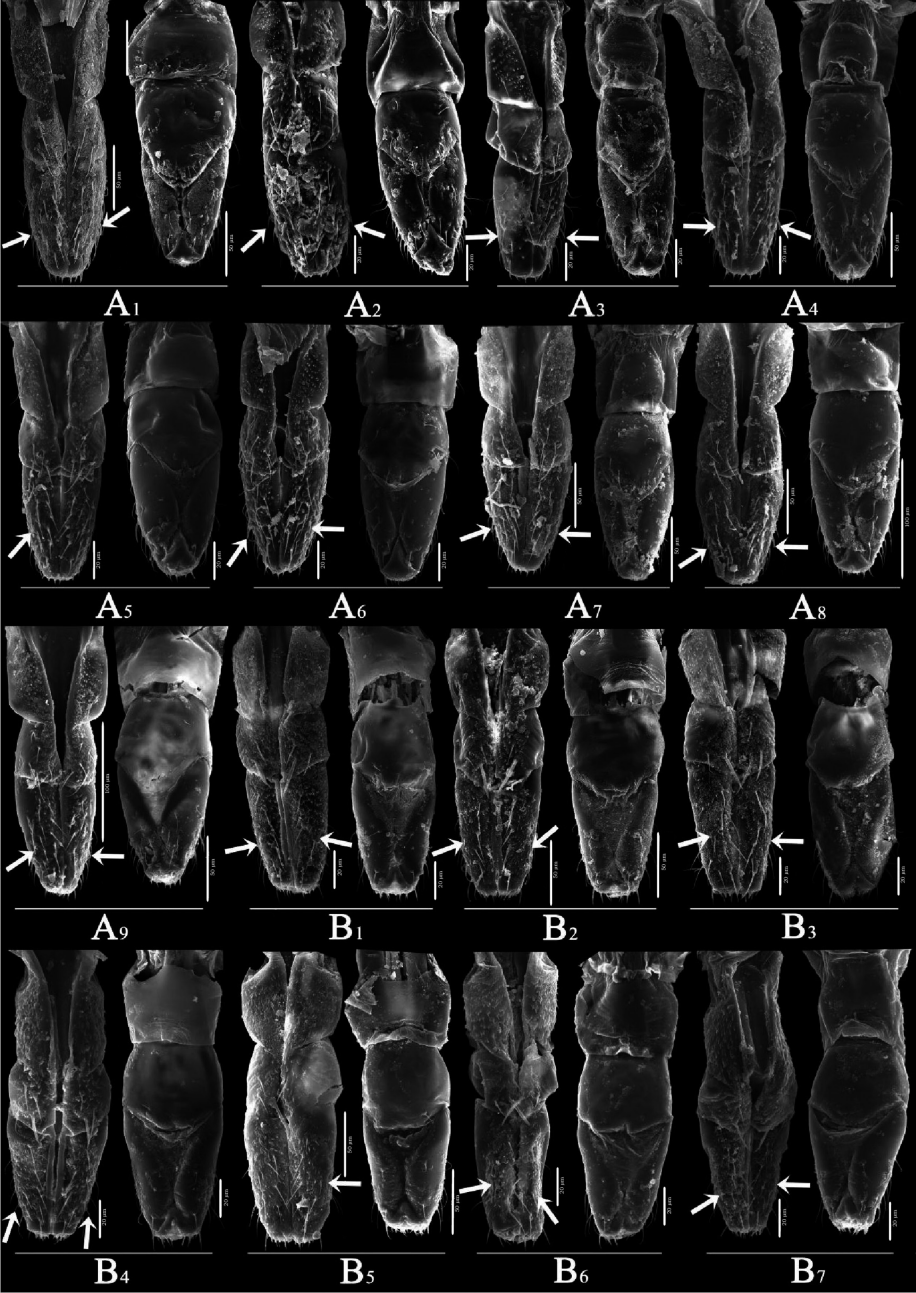
SEM observation of the labium (Lb) of Typhlocybine species (Left: Ventral view; Right: Dorsal view) (White arrow: SB) (a: *Singapora shinshana*; b: *Zyginella minuta*; 1, 2, 3….9: Different samples of the same species).

**FIGURE 12:**
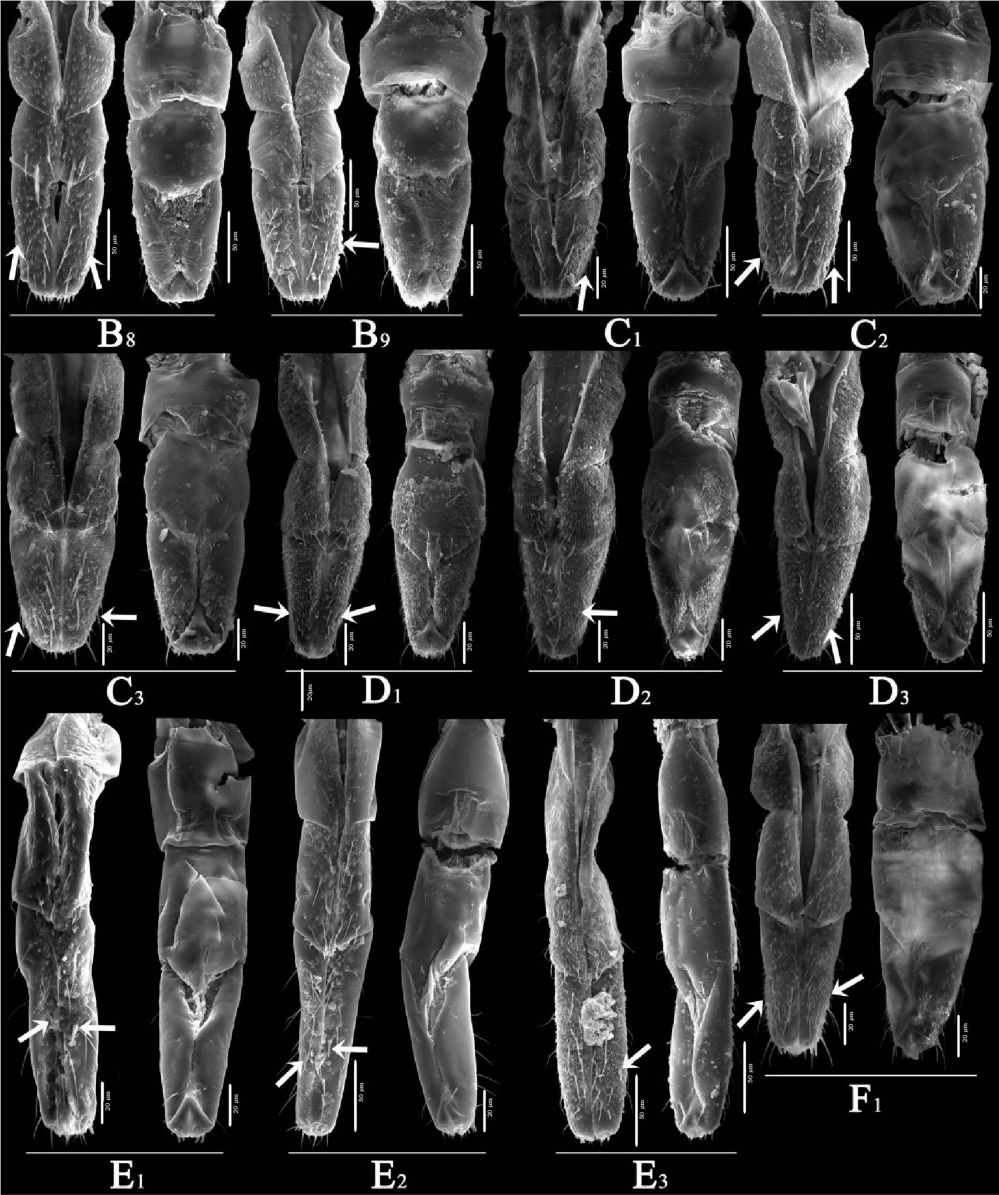
SEM observation of the labium (Lb) of Typhlocybine species (Continual figure of Figure 11) (Left: Ventral view; Right: Dorsal view) (White arrow: SB) (b: *Zyginella minuta*; c: *Arboridia remmi*; d: *Empoascanara sipra*; e: *Limassolla lingchuanensis*; f: *Alnetoidia dujuanensis*; g: *Asymmetrasca rybiogon*; h: *Empoasca* sp. ‐9; 1, 2, 3: Different samples of the same species).

**FIGURE 13:**
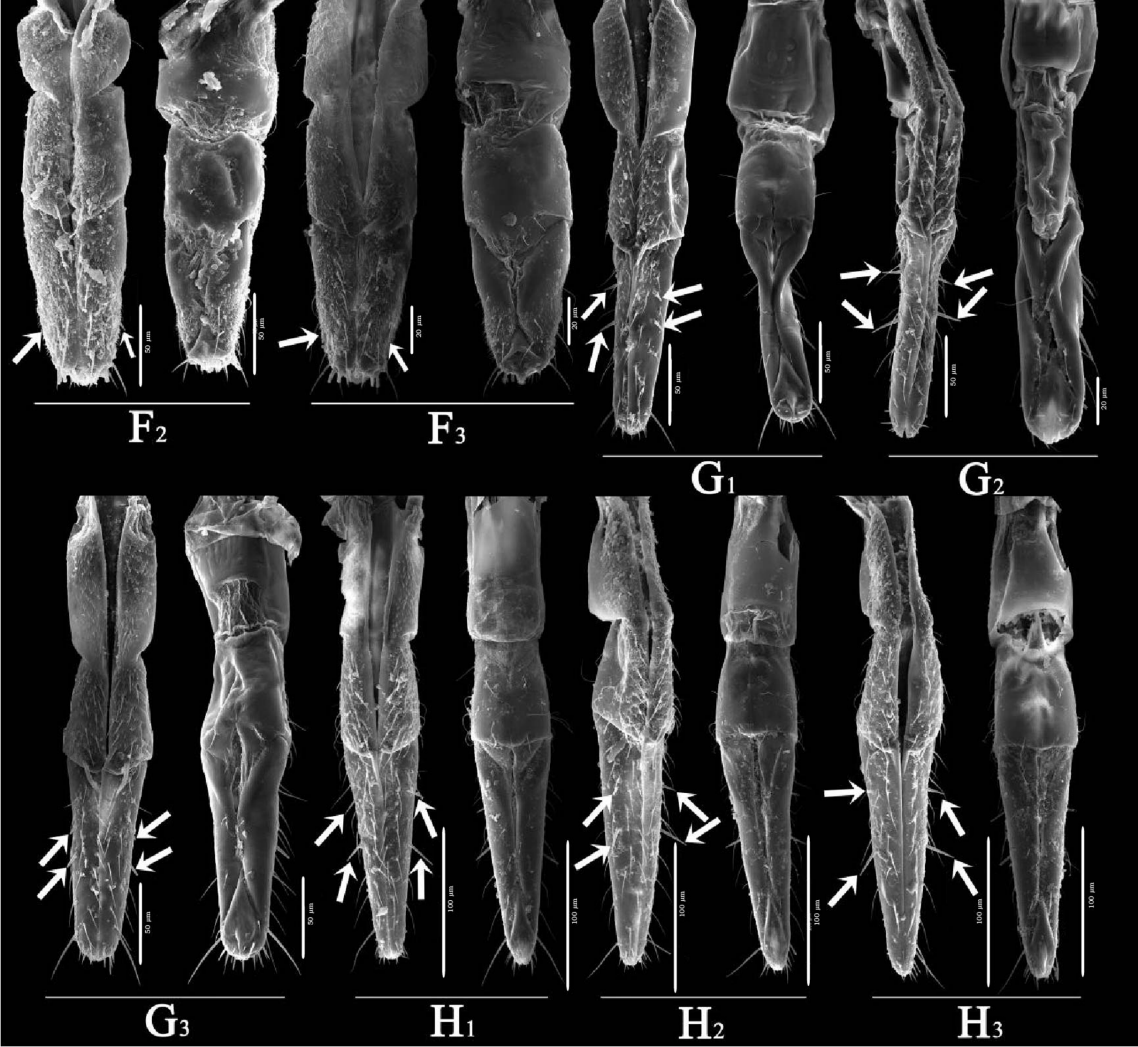
SEM observation of the labium (Lb) of Typhlocybine species (Continual figure of Figure 12) (Left: Ventral view; Right: Dorsal view) (White arrow: SB) (f: *Alnetoidia dujuanensis*; g: *Asymmetrasca rybiogon*; h: *Empoasca* sp. ‐9; 1, 2, 3: Different samples of the same species).

**FIGURE 14:**
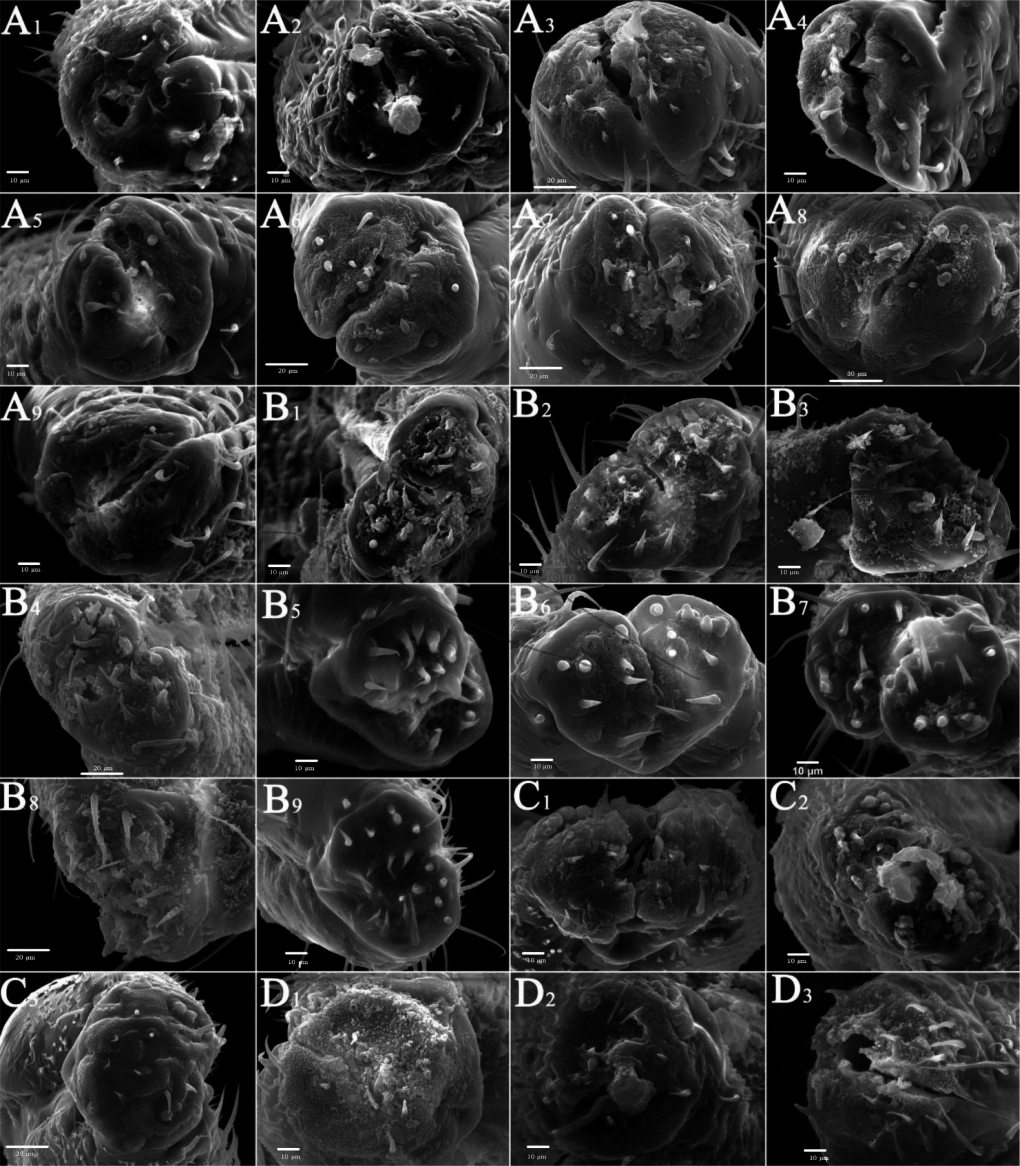
SEM observation of the tip of labium of Typhlocybine species (a: *Singapora shinshana*; b: *Zyginella minuta*; c: *Arboridia remmi*; d: *Empoascanara sipra*; 1, 2, 3….9: Different samples of the same species).

**FIGURE 15:**
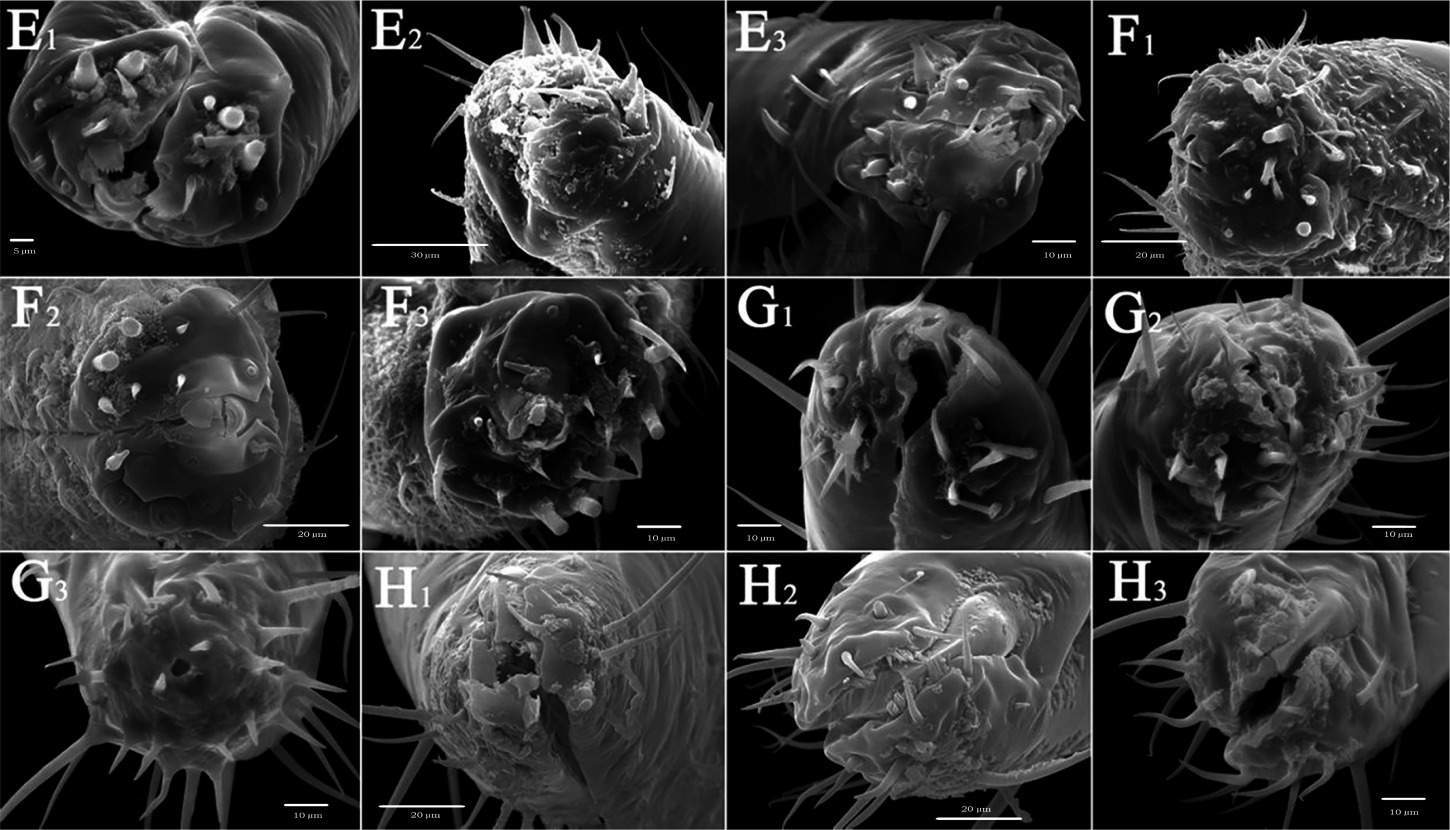
SEM observation of the tip of labium of Typhlocybine species (Continual figure of Figure 14) (e: *Limassolla lingchuanensis*; f: *Alnetoidia dujuanensis*; g: *Asymmetrasca rybiogon*; h: *Empoasca* sp. ‐9; 1, 2, 3: Different samples of the same species).

**FIGURE 16:**
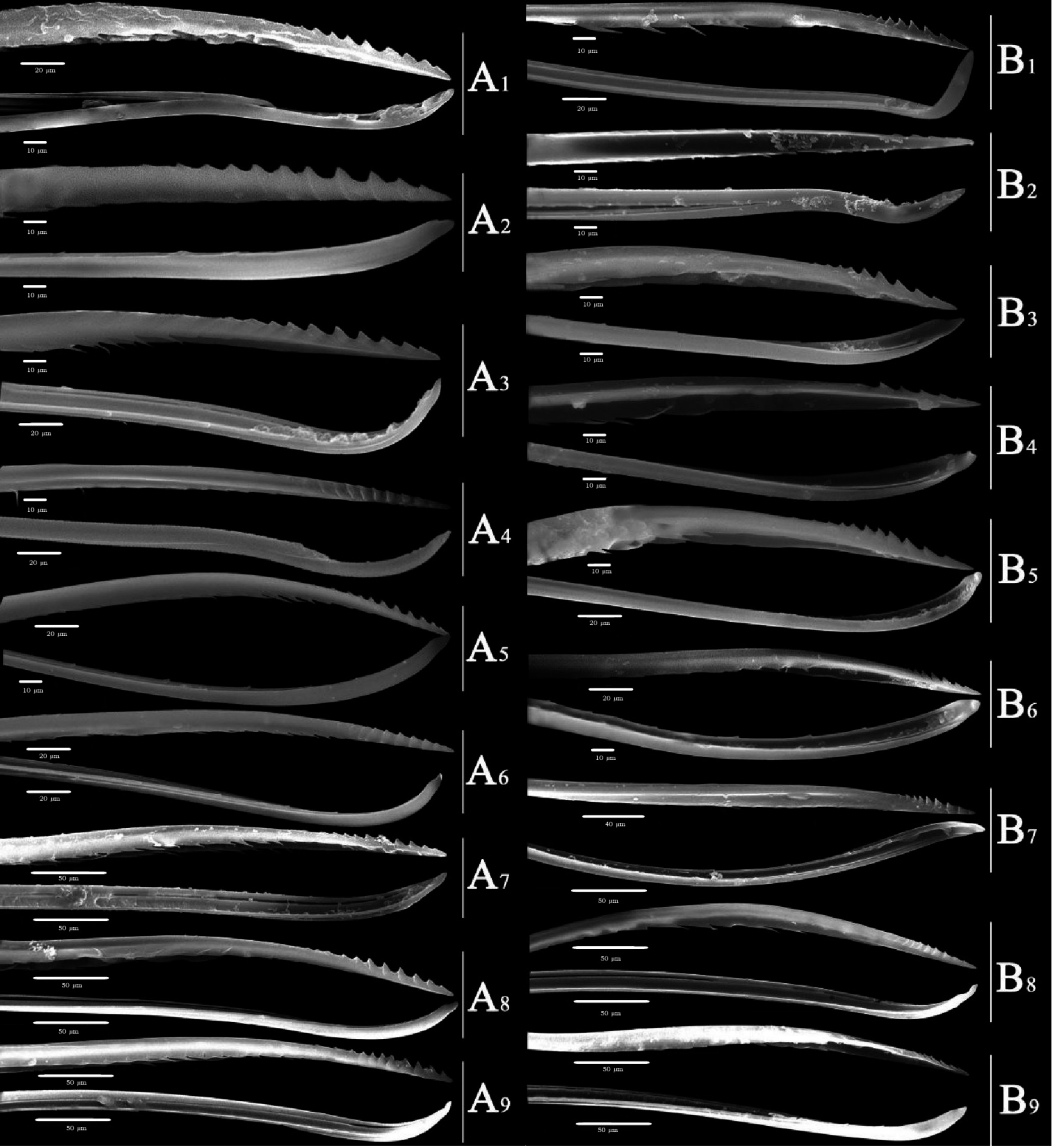
SEM observation of the stylet fascicle (Sf) of Typhlocybine species (Upper: Mandibular stylets) (Lower: Maxillary stylets) (a: *Singapora shinshana*; b: *Zyginella minuta*; 1, 2, 3….9: Different samples of the same species).

**FIGURE 17:**
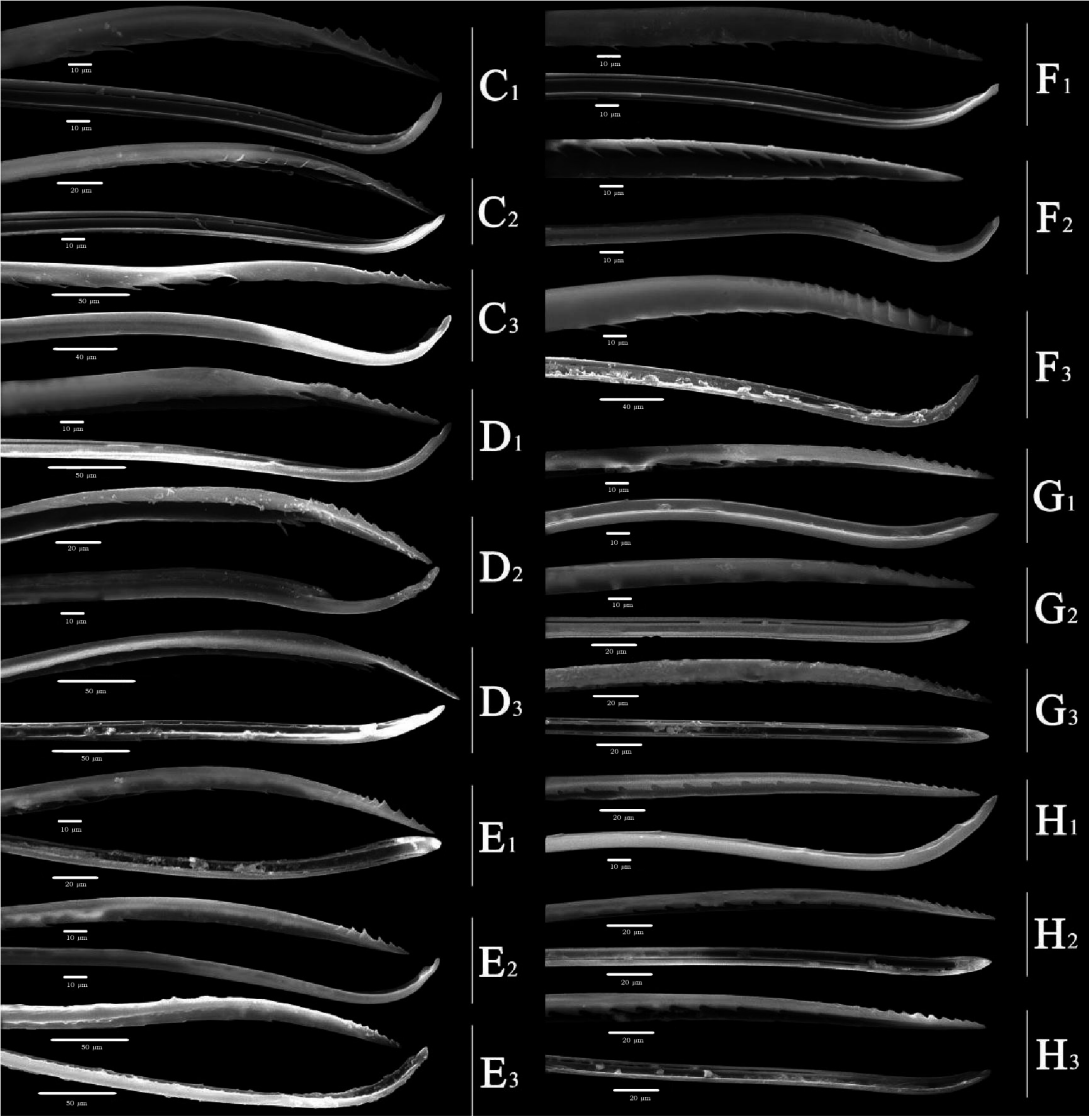
SEM observation of the stylet fascicle (Sf) of Typhlocybine species (Continual figure of Figure 16) (Upper: Mandibular stylets) (Lower: Maxillary stylets) (c: *Arboridia remmi*; d: *Empoascanara sipra*; e: *Limassolla lingchuanensis*; f: *Alnetoidia dujuanensis*; g: *Asymmetrasca rybiogon*; h: *Empoasca* sp. ‐9; 1, 2, 3: Different samples of the same species).

Overall (Figures 10, 11, 12, 13, 14, 15, 16, 17), the fine structures of the mouthparts of different species had certain interspecies differences, and the epidermal protrusions and types of sensilla tended to be consistent within the species (Table 4). Regarding the external form of the mouthparts, the labrum and labium of the two species of the tribe Empoascine (*A. rybiogon* and *E*. sp. ‐9) were relatively slender and similar in shape because they are closely related. Although the host plants of *S. shinshana* and *Z. minuta* differ, the morphology of the labium was similar, probably because the distances between the two habitats are similar. The stylets of all species were similar in morphology, and the mandibular stylets of different species were significantly different, which may be related to the plant parts they feed on.

**TABLE 4:** Comparison of the ultrastructure of mouthparts of 8 species of leafhoppers.

Species	Morphology											
	Labrum		Labium								Maxillary stylet	Mandibular stylet
	Length (μm)	Epidermal protrusion	Length (μm)	Lb‐1^a^		Lb‐2^b^		Lb‐3^c^		Lb‐tip^d^	Epidermal Protrusion (number)	Connective
				Length (μm)	Sensilla type (number)	Length (μm)	Sensilla type (number)	Length (μm)	Sensilla type (number)	Sensilla type (number)		
*Singapora shinshana*	72.16	Blunt	280–350	92.99	ST (2)	85.89	ST (15–24)	132.1	ST (52–66)/SB (2)	BSN (10–16)/OPSM (2–4)	Toothed/Ridged (10–11)	Obvious
*Zyginella minuta*	32.64	Blunt	210–270	74.83	C.p	70.26	ST (3–7)/OST (1–2)	96.82	ST (28–35)/SB (2)	BSN (10–12)/PGSU (6–8)	Toothed/Ridged (7–10)	Obvious
*Arboridia remmi*	55.26	Blunt	220–280	74.83	Mt./ST (2)	72.18	Mt./ST (15–17)	108.7	Mt./ST (26–31)/SB (2)	BSN (10–12)/PGSM (6–8)	Toothed/Ridged (6–8)	Not obvious
*Empoascanara sipra*	69.18	Blunt	250–300	86.01	Mt./ST (4)	79.54	Mt./ST (20–22)	109.6	Mt./ST (26–29)/SB (2)	BSN (12–16)	Toothed/Ridged (9–11)	Not obvious
*Limassolla lingchuanensis*	87.37	Blunt	290–360	86.23	Mt./ST (1–2)	96.92	ST (20–27)	149.6	ST (28–36)	BSN (8–10)/PGSM (6)	Toothed/Ridged (6–7)	Obvious
*Alnetoidia dujuanensis*	71.61	Blunt	250–290	73.57	Mt./ST (5–8)	83.61	Mt./ST (17–18)	112.8	Mt./ST (28–36)/SB (2)	BSN (12–14)/CLSU (3–4)	Toothed/Ridged (10–12)	Obvious
*Asymmetrasca rybiogon*	109.13	Blunt	370–500	137.29	Without	113.08	Mt./C.p/ST (30–38)	183.7	ST (42–50)/SB (4)	BSN (14–16)	Toothed/Ridged (9–10)	Obvious
*Empoasca* sp. ‐9	129.99	Blunt	440–550	145.37	C.p/ST (1–2)	121.85	Mt./C.p/ST (48–50)	223.5	ST (48–54)/SB (4)	BSN (12)	Toothed/Ridged (11–12)	Obvious

^a^ Lb‐1, First segment of Labium.

^b^ Lb‐2, Second segment of Labium.

^c^ Lb‐3, Third segment of Labium.

^d^ Lb‐tip, Tip of Labium.

From a temporal perspective, the number of sensilla was relatively high at the end of July and September and the number of sensilla was relatively low at the end of May, which is related to temperature differences and precipitation in the study areas. Spatially, the number of sensilla of *S. shinshana*, *A. dujuanensis*, and *E*. sp.‐9 was relatively constant, showing the following trend: Huajiang > Bijie > Shibing, which is related to the habitat difference in each region, the degree of rocky desertification. Therefore, we recognize that leafhopper species with higher densities of body sensilla are more abundant in rocky desertification areas where levels are high, which is considered an adjustment made by insects to adapt to deteriorating habitats. The unusually high number of *A. rybiogon* insects sampled in Bijie may be related to individual differences in the samples. In addition, the mouthparts from the other two study areas were shrunken, making it challenging to count the number of sensilla, so individual sample differences may have influenced quantity statistics. In addition to genetics, environmental factors play an important role in the quantity and distribution patterns of sensilla in leafhopper mouthparts.

### 3.2 Influence of habitat on the ultrastructure of the typhlocybine body surface

Antennae and mouthparts are the main organs used by leafhoppers to select host plants. Leafhoppers use chemoreceptors to detect plant volatiles for host plant selection and orientation, and mechanoreceptors to sense vibrations on the surface of host plants and determine the impact of other organisms on the habitat, such as biological invasion, wind, and rain, thus adjusting the insect's position on the plant surface (Rani & Madhavendra, 2005). Our results indicate that typhlocybine leafhopper species having higher densities of body sensilla are more abundant in hotter, drier habitats, while those with lower densities predominate in cooler, wetter habitats, which is consistent with our speculation in the Introduction. At the three study sites, the vegetation in July and September was lusher and more diverse than in May, and other biological activities were more active. For a better selection of host plants and perception of changes in the external environment during this period, more sensilla may appear on the body surface of typhlocybine species to help them adapt better to environmental changes.

According to the distribution of the sensilla of typhlocybine insects (Table S7), we removed the sensory data distributed only on a small number of individuals (<1/2) and used the remaining data and the corresponding environmental factors for redundancy analysis. The results are as follows (Table 5).

**TABLE 5:** The interpretation rate of environmental factors on the number of leafhopper surface sensilla.

Region/time	Interpretation rate						
	First axis (%)	Second axis (%)	Third axis (%)	Fourth axis (%)	Front two axles (%)	Total (%)	Mainly influence factors
Study area	62.02	21.06	9.14	4.75	83.09	96.97	BL BW LWC
Huajiang	61.16	25.45	8.13	2.73	86.61	97.47	PD BW LWC
Bijie	64.08	21.34	8.65	3.29	85.42	97.36	BL BW
Shibing	51.47	24.25	18.44	2.68	75.72	96.84	BL BW
End of September	69.70	16.48	8.23	2.86	86.18	97.28	PD BL BW LWC
End of May	63.53	20.50	8.53	3.54	84.03	96.11	BL BW
End of July	50.38	25.04	19.25	2.84	75.43	97.52	BL BW

**FIGURE 18:**
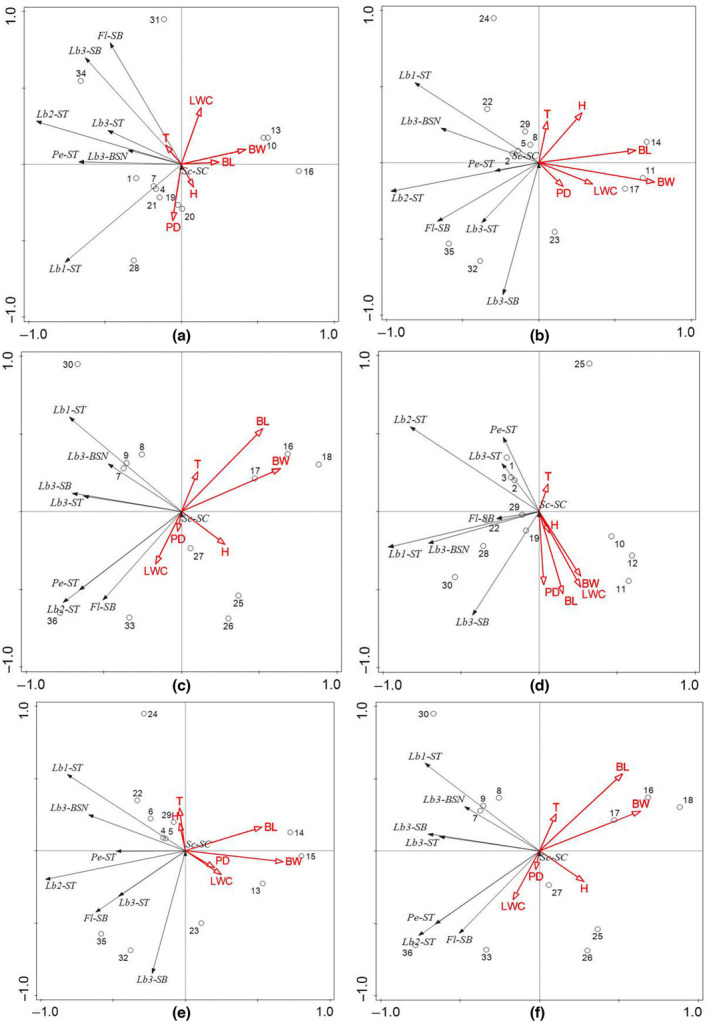
Redundancy analysis between the sensilla number on Typhlocybinae body surface and the environmental factors. (a) Huajiang. (b) Bijie. (c) Shibing. (d) end of September. (e) end of May. (f) end of July (Sc‐SC: The sensilla chaetica on the Scape; Pe‐ST: The sensilla trichodea on the Pedicel; Fl‐ST: The sensilla basiconica on the Fiagellum; Lb1‐ST: The sensilla trichodea the first section of Labium; Lb2‐ST: The sensilla trichodea on the second section of Labium; Lb3‐ST: The sensilla trichodea on the third section of Labium; Lb3‐SB: The sensilla basiconica on the third section of Labium; Lb3‐BSN: The Cone‐like sensilla on the third section of Labium. temperature (T), humidity (H), Precipitation days (PD), blade length (BL), blade width (BW), leaf water content (LWC). The numbered circles: The number of different types of sensilla in different body parts).

**FIGURE 19:**
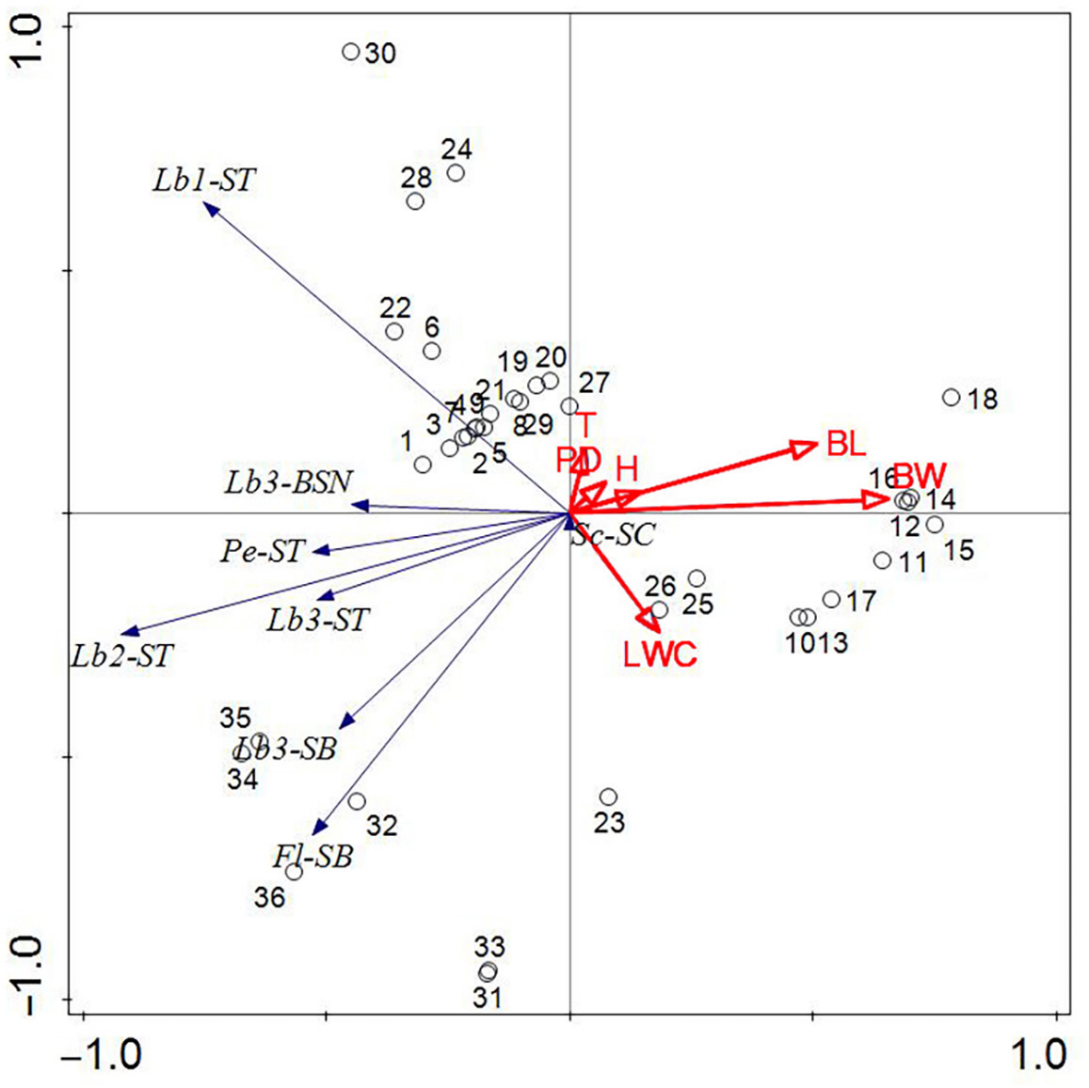
Redundancy analysis between the sensilla number on Typhlocybinae body surface and environmental factors in study areas (Sc‐SC: The sensilla chaetica on the Scape; Pe‐ST: The sensilla trichodea on the Pedicel; Fl‐ST: The sensilla basiconica on the Fiagellum; Lb1‐ST: The sensilla trichodea the first section of Labium; Lb2‐ST: The sensilla trichodea on the second section of Labium; Lb3‐ST: The sensilla trichodea on the third section of Labium; Lb3‐SB: The sensilla basiconica on the third section of Labium; Lb3‐BSN: The Cone‐like sensilla on the third section of Labium. temperature (T), humidity (H), Precipitation days (PD), blade length (BL), blade width (BW), leaf water content (LWC); The numbered circles: The number of different types of sensilla in different body parts).

From a spatial perspective (Figure 18a–c), the number of sensilla on the antennae and mouthparts of typhlocybine insects in the Huajiang and Bijie study areas was relatively large, whereas that in the Shibing study area was relatively small. In terms of time (Figure 18d–f), the number of sensilla on the antennae and mouthparts of the leafhoppers in the study areas at the end of July and September was significantly correlated with environmental factors, whereas the number was relatively small at the end of May. For the entire study area (Figure 19), the cumulative interpretation rate of the four axes reached 96.97%. This shows that environmental factors have a considerable impact on the number of sensilla on the body surface of typhlocybine insects. Among them, the correlation between BL, BW, and LWC and the sensilla number of typhlocybine insects was greater, and the data mentioned above are all relevant indicators of the host plants. Among these environmental factors, the growth and development of the host plant are important factors that affect the number of surface sensilla in leafhoppers. Insects can receive gas compounds released by various plants through their body surface sensilla, looking for suitable host plants in the habitat after the identification of host plants, exploratory feeding, selection of habitat, spawning grounds, and other activities. Therefore, changes in the host plant greatly affect the number of sensilla on the insect's body surface.

## 4 DISCUSSION

Observations revealed that the antennae of typhlocybine insects were all setaceous, similar to those of other known leafhoppers. The scape has two sensilla chaetica, the pedicel has sensilla trichodea, and the flagellum has at least one sensilla basiconica. Leafhopper species exhibit different types of antennal epidermal protrusions (Aljunid & Anderson, 1983; Gao et al., 2002; Guo et al., 2018; Liang & Fletcher, 2002; Stacconi et al., 2009; Stacconi & Romani, 2012). Previous studies have shown that leafhopper pedicels have several scaly structures. Researchers have identified such structures in the following species: *Taurotettix elegans* (Melichar, 1900), *Empoasca onukii* (Matsuda, 1952), *Psammotettix striatus* (Linnaeus, 1758), *Empoasca vitis* (Göthe, 1875), *Scaphoideus titanus* (Ball, 1932), *Exitianus indicus* (Distant, 1908), *Hecalus nitobei* (Matsumura, 1912), and *Nephotettix cincticeps* (Uhler, 1896) (Dai et al., 2011; Ni & Dai, 2010; Qiao et al., 2016; Stacconi & Romani, 2012; Su et al., 2015; Zhang et al., 2011). However, in the present study, the antennal surface of *E. sipra* (Dworakowska, 1980) showed significant differences; the pedicel was covered with microtrichia instead of scaly structures. The flagella of leafhoppers of different species differ in morphology, and the number of segments is quite different. The flagellum of typhlocybine species in this study usually had 7–18 segments, and most of the flagellum was segmented only in the first half; however, Guo et al. (2018), in their study of the antennae morphology of adult *Chlorotettix nigromaculatus* (DeLong, 1923), found that its flagellum is segmented from head to tail, with approximately 60–64 subsegments (Guo et al., 2018).

The mouthparts of typhlocybine insects are similar in structure to those of other insects of Hemiptera but have different fine structures (Anderson et al., 2006; Jin, 2012; Tavella & Arzone, 1993; Wiesenborn, 2004). The labrum is triangular with smooth surfaces or protrusions, and there are certain differences in the morphology of epidermal protrusions between different species. In this study, the labrum of typhlocybine insects had no sensilla, but Pan (2013) found that the labrum of *Cicadella viridis* (Linnaeus, 1758) and *Atkinsoniella grahami* (Young, 1986), which belong to Cicadellidae, had a coeloconic sensilla distribution (Pan, 2013). There are two types of labium, stubby and slender, with smooth surfaces or protrusions. There are often more sensilla trichodea distributed on the labium ventral surface and the most symmetrical distribution along the labial groove. Most species had at least one pair of sensilla basiconica symmetrically distributed along the labial groove in the third labial segment. The number and size of sensilla, epidermal protrusions, and Mt on the surface of the labium can vary with developmental stage (Hao et al., 2016; Leopold et al., 2003). Five different types of sensilla–BSN, CLSU, PGSU, PGSM, and OPSM–were found on top of the labium of typhlocybine insects, and BSN was widely distributed on top of the labium in various species. According to existing research, these sensilla are speculated to be mechanical and chemical sensilla that can sense the temperature, humidity, and surface information of host plants, etc. (Cobben, 1978, 1979; Schoonhoven & Henstra, 1972; Slifer et al., 1959). Boyd et al. (2002) proposed that the shape of the end of the mandibular stylet of insects is related to the plants they feed on (Boyd et al., 2002). This study found that the structure of the stylets was not very different and that there was no special structure.

Previous studies have not investigated the relationship between insect body surface ultrastructure and environmental factors. In this study, the relationship between the number of sensilla on the antennae and mouthparts of typhlocybine insects and environmental factors was discussed. In addition to kinship, the host plant was the main factor affecting the number of sensilla on the antennae and mouthparts of typhlocybine insects.

Regarding the spatial distribution, the influence of the environment on the surface sensilla of leafhoppers in the three study areas was as follows: Huajiang > Bijie > Shibing, which is consistent with the rocky desertification classification grade. In the Huajiang study area, rainfall days and host plants mainly influenced the occurrence and development of leafhopper sensilla. This may be because the rock desertification level in the Huajiang area is the highest and the environment is the most severe, with high temperatures, low precipitation, large terrain changes, small vegetation coverage, and more soil erosion. Consequently, leafhopper species living in this area require more receptors on their body surface to sense changes in the external environment and meet their survival needs (Liu et al., 2021; Yin et al., 2020). The degree of rocky desertification in the Bijie study area is medium and the terrain is high. Soil erosion is severe, and the original vegetation in the area is heavily damaged by human production and construction activities (Li et al., 2020; Liu, Zhang, et al., 2020; Ren et al., 2020). However, the rainfall in this area is sufficient, and vegetation restoration is better than that in Huajiang under comprehensive ecological management. Therefore, the number of sensilla on the leafhoppers in this study area was mainly influenced by the growth and development of the host plants. The Shibing study area is a non‐potential rocky desertification area and is a World Natural Heritage Site. The internal hydrothermal conditions are well coordinated, forest protection is good, the soil layer is thick, the vegetation is lush, and the ecosystem's self‐regulating ability is strong (Cai et al., 2019; Dai et al., 2021). Therefore, the number of sensilla on leafhoppers was mainly affected by the seasonal growth and development of the host plants.

In terms of time distribution, the influence of the ecological environment on the diversity of the leafhopper community over the 3 months was as follows: end of September > end of May > end of July, and was mainly affected by habitat characteristics during the three different periods. At the end of July, the number of sensilla on the antennae and mouthparts of leafhoppers in the study areas had the most significant relationship with environmental factors, which were mainly affected by host plants. Therefore, it is not difficult to see that in summer, the vegetation is lush, and the temperature, humidity, and precipitation days vary widely, but the high‐temperature period is long and suitable for the growth of leafhoppers. Plant transpiration is high, and many plants release large amounts of volatile compounds. Therefore, leafhoppers increase the number of surface sensors to better locate and feed on host plants (Han et al., 2021; Li et al., 2022; Zhao et al., 2012). At the end of September, the number of sensilla on the antennae and mouthparts of the leafhoppers in the study areas was strongly related to environmental factors and was mainly affected by precipitation and host plants. A subtropical monsoon wet climate prevails in most areas of Guizhou. In autumn, the light is sufficient, but the number of rainy days increases, and the vegetation begins to languish, which not only affects the location selection of insects on plant surfaces and the search for a safe habitat but also affects the surface sensilla for the reception of plant volatiles and positioning direction (Guo et al., 2018; Rani & Madhavendra, 2005; Zhao et al., 2012). At the end of May, the number of sensilla on the antennae and mouthparts of the leafhoppers in the study areas was less related to environmental factors and was mainly influenced by host plant growth and development. This period is in late spring, and Guizhou Province is influenced by the plateau climate (Guizhou People's Publishing House, 2001; Yan, 2010). The temperature in most areas is low, and the growth and development of plants are sluggish, affecting the growth and development of leafhoppers, which in turn affects the number of sensilla on the body surface of leafhoppers.

As insects choose a specific habitat according to their food preferences (Jauker et al., 2009), leafhoppers locate their habitat and food sources, that is, host plants, using their sensilla. This is one of the principal environmental factors that influence leafhopper populations. Insect sensilla result from the combined effects of natural selection and environmental interactions (Na et al., 2008b). On the same host plant, other external environmental factors had a greater effect on the surface sensilla of leafhoppers. With a simpler living environment and fewer natural enemies, the type of antennal sensilla is simpler and the number of sensilla is lower.

## 5 CONCLUSIONS

In this study, the surface ultrastructural morphology of typhlocybine leafhoppers on different plants in three karst rocky desertification areas of Guizhou was investigated. The number of sensilla on the surface of typhlocybine insects and its correlation with the geographic environmental characteristics in different rocky desertification areas were determined. The results showed that there were differences in the number and type of body surface sensors of leafhoppers in different grades of rocky desertification in different ecological environments. Therefore, as ecological indicators of insects, changes in the type and number of leafhoppers sensilla can reflect the evolution of the regional geographical environment and provide a reference for ecological restoration in rocky desertification areas. The present study revealed, to a certain extent, the response of leafhoppers to changes in the surrounding environment, and the biotic and abiotic factors affecting insect ultra‐morphology require further exploration and research.

## AUTHOR CONTRIBUTIONS

**Jiajia Chen:** Investigation (equal); writing – original draft (equal); writing – review and editing (equal). **Jia Jiang:** Conceptualization (equal); methodology (equal); writing – original draft (supporting); writing – review and editing (supporting). **Ni Zhang:** Supervision (equal); validation (equal). **Yuehua Song:** Funding acquisition (equal); project administration (equal); resources (equal).

## FUNDING INFORMATION

This work was supported by the World Top Discipline Program of Guizhou Province: Karst Eco environment Sciences (No. 125 2019 Qianjiao Keyan Fa), the Innovation Group Project of Education Department of Guizhou Province ([2021]013), the National Natural Science Foundation of China (32260120), the Natural Science Foundation of Guizhou Province (Qiankehejichu‐ZK [2023] General 257) and the Training Program for High‐level Innovative Talents of Guizhou Province (QiankehepingtairencaiGCC[2023]032).

## CONFLICT OF INTEREST STATEMENT

The authors declare no conflict of interest.

## Supporting information


Table S1

Table S2

Table S3

Table S4

Table S5

Table S6

Table S7

## Data Availability

All data generated or analyzed in this study are included in this article and its Supporting Information files. Supplementary Data are available on the Knowledge Network for Biocomplexity (https://knb.ecoinformatics.org/view/doi:10.5063/F1QV3K0R).
